# Highly elevated polygenic risk scores are better predictors of myocardial infarction risk early in life than later

**DOI:** 10.1186/s13073-021-00828-8

**Published:** 2021-01-28

**Authors:** Monica Isgut, Jimeng Sun, Arshed A. Quyyumi, Greg Gibson

**Affiliations:** 1 School of Biological Sciences, Georgia Institute of Technology, 950 Atlantic Drive, EBB1 Suite 2115, Georgia Tech, Atlanta, GA 30332 USA; 2 Department of Computer Science, University of Illinois Urbana-Champaign, Champaign, USA; 3 Department of Medicine, Emory Clinical Cardiovascular Research Institute, Emory University School of Medicine, Atlanta, USA

**Keywords:** Coronary artery disease, Ischemic heart disease, Myocardial infarction, Polygenic risk scores, Risk assessment

## Abstract

**Background:**

Several polygenic risk scores (PRS) have been developed for cardiovascular risk prediction, but the additive value of including PRS together with conventional risk factors for risk prediction is questionable. This study assesses the clinical utility of including four PRS generated from 194, 46K, 1.5M, and 6M SNPs, along with conventional risk factors, to predict risk of ischemic heart disease (IHD), myocardial infarction (MI), and first MI event on or before age 50 (early MI).

**Methods:**

A cross-validated logistic regression (LR) algorithm was trained either on ~ 440K European ancestry individuals from the UK Biobank (UKB), or the full UKB population, including as features different combinations of conventional established-at-birth risk factors (ancestry, sex) and risk factors that are non-fixed over an individual’s lifespan (age, BMI, hypertension, hyperlipidemia, diabetes, smoking, family history), with and without also including PRS. The algorithm was trained separately with IHD, MI, and early MI as prediction labels.

**Results:**

When LR was trained using risk factors established-at-birth, adding the four PRS significantly improved the area under the curve (AUC) for IHD (0.62 to 0.67) and MI (0.67 to 0.73), as well as for early MI (0.70 to 0.79). When LR was trained using all risk factors, adding the four PRS only resulted in a significantly higher disease prevalence in the 98th and 99th percentiles of both the IHD and MI scores.

**Conclusions:**

PRS improve cardiovascular risk stratification early in life when knowledge of later-life risk factors is unavailable. However, by middle age, when many risk factors are known, the improvement attributed to PRS is marginal for the general population.

**Supplementary Information:**

The online version contains supplementary material available at 10.1186/s13073-021-00828-8.

## Background

Ischemic heart disease (IHD) is the leading cause of death worldwide and affects more than 110 million people globally [1, 2]. Although the age-adjusted prevalence of IHD has been declining recently in the USA [3], its global prevalence continues to increase [2]. Several risk prediction models for IHD have been developed, and these incorporate a number of demographic, behavioral, lifestyle, and clinical risk factors as covariates for prediction [4, 5]. Some of the most well-known models are time-to-event scores, such as the Framingham Risk Score [6], the European SCORE [7], and the ACC (American College of Cardiology)/AHA (American Heart Association) pooled cohort equation (PCE) [8]. These models can be improved in several ways. First, there is evidence that individuals with low 10-year Framingham IHD risk in middle age tend to have a similar lifetime risk to those with medium and high 10-year risk [9], which suggests that using prevalent risk models along with time-to-event models in risk prediction can be important. Moreover, research also argues that including non-conventional biomarkers in risk models (e.g., apolipoproteins, C-reactive protein), as was done in the Reynolds Risk Score for women [10] and in a recent machine learning-based prediction algorithm [11], can improve prediction. This motivates the potential for inclusion of genetic data in IHD risk models, as a baseline lifetime risk estimate, though we emphasize that the prevalence estimates in the UK Biobank convenience sample used in our study are not truly lifetime, as some individuals will experience heart attacks or attain a diagnosis of heart disease at some point in the future, while others will have passed away prior to enrolment.

Genome-wide association studies (GWAS) have enabled the development of several polygenic risk scores (PRS) for IHD risk prediction. PRS consist of a selection of genomic variants and their associated GWAS-derived weights (effect sizes) for a condition of interest [12, 13]. They can be used to predict individuals’ disease risks based on their genotypes for each of the selected variants. The premise behind the use of PRS is that much of the genetic risk for many common adult-onset diseases is attributed to the cumulative effect of many common variants with small effect sizes rather than rare variants with large effect sizes [14]. PRS have become popular recently, and have been developed for various diseases, including breast cancer [15], mental health [16], inflammatory bowel disease [17], and many more.

PRS can potentially play an important role in the primary prevention of IHD, as they allow risk to be assessed at the time of birth and can thus guide the targeting of interventions early in life. Nonetheless, the adoption of these scores in routine clinical practice remains in its infancy [18]. An important unresolved issue is what proportion of the population may benefit from PRS assessment. Following [19], we take the view that the greatest impact is likely to be seen at the tails of the distribution, typically the top few percent, where threefold or more elevated risk relative to the population mean approximately corresponds to the effect of monogenic variants that are already regarded as clinically actionable. Sensitivity is necessarily low with fewer than 10% of cases recalled in these upper percentiles, and correspondingly precision may also be in the same range, but targeting treatment to just 3% of individuals could conceivably prevent 10% or more of cases if they are also particularly responsive [20]. This can also dramatically reduce the number needed to treat. Conversely, in the smallest percentiles, very low risk individuals may be spared expensive and potentially harmful medications [21]—though that is not a concern for standard blood pressure and cholesterol lowering drugs.

It is also unclear to what extent PRS enhance heart disease risk assessment when combined with lifestyle and clinical risk factors that are commonly used in traditional risk scores. Inouye et al. [22] showed that a metaGRS polygenic score performs very differently as a function of the number of conventional risk factors, so much so that the cumulative risk of CAD between the ages of 45 and 75 is very similar for individuals in the bottom quintile of polygenic risk but who have two risk factors and for individuals with no risk factors in the top quintile of polygenic risk. They concluded that “the predictive ability of the metaGRS was largely independent of established risk factors for CAD, implying that genetic information complements conventional risk factors.” By contrast, two recent studies by Mosley et al. [23] and Elliott et al. [24] came to the conclusion that PRS do not contribute substantially to prediction accuracy when data on traditional risk factors is available. Both studies incorporated PRS into 10-year incident heart disease risk prediction algorithms using Cox proportional hazards regression modeling incorporating the ACC/AHA pooled cohort equations (or the QRISK3 score [25] used in the UK) and various polygenic risk scores. The performance of the combined models was compared to that of the original 10-year scores in the relatively small Atherosclerosis Risk in Communities (ARIC) and Multi-Ethnic Study of Atherosclerosis (MESA) longitudinal cohorts [23] and UK Biobank (UKB) [24], the latter after training on prevalent cases. A third study by Mars et al. [26] similarly found no significant improvement in C-index when a PRS was added to the ASCVD in the 20,000-person FinnRisk cohort (0.823 versus 0.820), while the net reclassification index (NRI) only improved a few percent for early onset cases and late onset controls.

Here, providing a complementary analysis to the above-mentioned incident case studies, we generated several prevalent IHD risk prediction models by training a logistic regression algorithm on ~ 440K European individuals in the UKB dataset using as features different combinations of conventional risk factors, with and without including four IHD-specific PRS [19, 22, 27, 28]. The features used in these models were extracted from the UKB questionnaire, Hospital Episodes Statistics (HES), and genotype datasets. They were categorized as PRS, set-at-birth (biological sex, ancestry), and non-fixed (birth year as a proxy for age, body mass index, blood pressure, the combination of serum triglycerides, cholesterol and LDL to capture lipidemia, smoking status, diabetes status, and family history) based on whether they are set at birth or change over time. Birth year (as a proxy for age) was included in the non-fixed category because it changes over time and becomes a risk factor later in life. In addition to IHD, we also generated models using myocardial infarction (MI) [29, 30] and first MI at or before age 50 (early MI) as prediction labels.

This study expands on previous findings in several ways. First, to determine to what extent the results of the previous studies of 10-year (incident) risk can be generalized to lifetime (prevalent) risk prediction, which has been suggested to be complementary to time-to-event risk prediction, we focused our analysis on prevalent risk prediction using logistic regression. Second, to assess the degree to which the results are consistent across different phenotype definitions, we ran all experiments separately using three different heart disease phenotype definitions (IHD as the broader definition and MI and early MI as sub-categories of IHD). Elliott et al. [24] considered a variety of case definitions, but we expand the analysis to include early MI which, having an earlier age of onset, may imply more of a genetic basis for risk prediction as compared with other lifestyle risk factors. Third, we incorporated multiple PRS together, rather than just one, in the risk prediction models. This allowed for a more generalized comparison between PRS and traditional risk factors compared to including just one PRS and provides insight into the range of genetic risk over which PRS may add predictive value. Fourth, to generalize the results across ancestries, we ran all analyses both on the full UK Biobank and on the subset of European-only individuals. Since risk score distributions differ between ancestry groups, we evaluated the ability of logistic regression (LR) modeling to adjust for this bias which otherwise generates prevalence-risk profiles in which the highest genetic-risk percentiles have reduced prevalence. Finally, an additional goal of the study was to compare estimated prevalent risk for IHD, MI, and early MI between propensity-matched subpopulations of individuals in the UK Biobank based on blood pressure and statin medication intake. Considering all of these perspectives together with a comparison of the clinical features of individuals with higher total than genetic risk, we infer that polygenic risk assessment really is not independent of conventional risk from middle age since much of the genetic component is regulating those known risk factors.

## Methods

### Sample selection

The population sample consisted of the set of individuals in the UK Biobank (UKB) study for whom genotype and binary sex data were available [31]. Approximately 50,000 samples were genotyped using the UK BiLEVE Axiom array, and the remaining ~ 450,000 samples were genotyped using the UK Biobank Axiom array. All samples were imputed by the UK Biobank [31], resulting in a total dataset of ~ 92.6 million variants in 487,442 individuals accessed August 2018. Out of these, a total of 62 individuals with missing sample IDs or unknown sex were excluded, for a final sample size of 487,380 individuals of all ancestries.

For the primary analyses, a genetically more homogeneous subset of “British and Irish with European Ancestry” individuals was extracted, based on the combination of self-reported ancestry (White British and Irish) as well membership in a cluster of European-ancestry individuals defined by the first two genetic principal components of the entire UK Biobank sample. This subset of 441,173 individuals indicated by blue crosses in Additional file 1: Fig. S1 was identified by performing k-means clustering on the first two principal components of the genotype matrix for the entire UK Biobank, with *k* = 4 identifying four major ancestry groups. Intersection with the self-reported White British (472,233 individuals) and White Irish (14,003) individuals from data field 21,000 of the UK Biobank registry, resulted in the final sample. All analyses were thus conducted twice—one time with the 441,101 cohort of British and Irish European individuals only and once again with the 487,380 cohort of UK Biobank individuals of all ancestries.

### Polygenic risk score calculations

Four PRS previously developed for IHD risk prediction were used in this analysis. The scores were somewhat independently developed and, though correlated (*R* ranging between 0.12 and 0.67), are expected to capture slightly different components of genetic risk and collectively to provide further enhanced prediction. Each PRS consists of a list of single-nucleotide variants and weights derived from GWAS studies. Scores for each sample individual were generated using Plink 2.0 software [32], which calculates a weighted sum of allele dosage by GWAS weight for each variant. The “FDR202” score consists of 202 common variants with a false discovery rate *q* value of < 0.05 that originate from the CARDIoGRAM consortium 1000 Genomes GWAS meta-analysis [22]. Of these 202 variants, we used 194 that were available in the UKB imputed genotypes database to calculate the PRS. The 1.7M score [22] consists of approximately 1,745,180 variants and their weights from the same GWAS, of which 1,465,932 were available in the UKB database. The GRS46K score consists of 46,773 variants identified by Abraham et al. [22, 28] from stage 2 of the CARDIoGRAM consortium metabochip GWAS [33], of which 45,498 were available in the UKB database. The 6M score consists of 6,630,150 variants identified by Khera et al. using the LDPred algorithm [34], of which 6,629,369 were available in the UKB database [31].

### Logistic regression labels

**Fig. 1:**
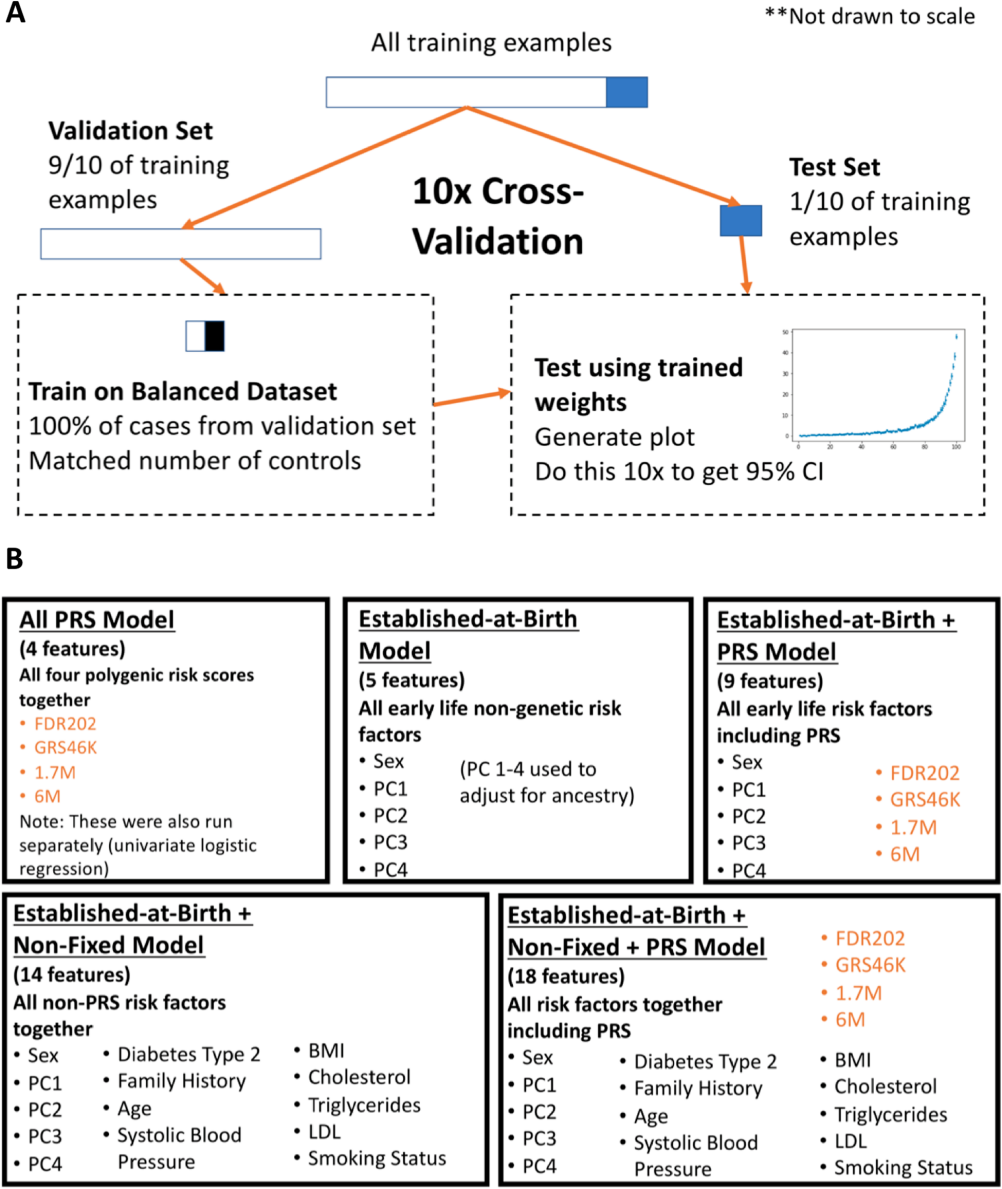
Cross-validation scheme for logistic regression training and testing. **a** Schema indicating how each risk score was generated using a logistic regression algorithm trained with a selection of variable features and a dependent outcome label (IHD, MI, early MI). Ten folds of cross-validation were performed after dividing the full UKB dataset into ten equal subsets, each of which was iteratively used as a test set (1/10 of the data) after training on the others (9/10 of the data). In each iteration of the training, all cases were matched with an equal number of randomly sampled controls. **b** List of features included in each of five different LR models

A total of 14 input features were extracted from the UKB Hospital Episode Statistics (HES) and questionnaire datasets for inclusion in this analysis. We selected as features conventional IHD risk factors that are already included in several widely used risk scores, for which UKB data was available. Note that inclusion of additional non-genetic attributes such as diet, medication, and lifestyle factors would be expected to improve model performance further [11], while also reducing the impact of the PRS component. Using the logistic regression framework, we generated five different risk prediction models, each with a different subset of features as summarized in Fig. 1a: (a) PRS only (4 features), (b) established-at-birth only (5 features), (c) established-at-birth including PRS (9 features), (d) non-fixed and established-at-birth without PRS (14 features), and (e) non-fixed and established-at-birth including PRS (18 features full model). Some features were categorical while others were continuous, and missing data for these categories were handled as indicated in Additional file 2: Table S1. To represent residual components of ancestry in the White British subset of the UK Biobank, we used as features the first four genotypic principal components (PC1, PC2, PC3, PC4) [35, 36] (Additional file 1: Fig. S1), with biological sex as the third established-at-birth feature. The same four PCs were also included to capture ancestry in the secondary analysis of the full UKB.

For prediction, all individuals were given a binary (1,0) label for prevalent case or control status, respectively, for each of the three conditions of interest (IHD, MI, and early MI). An individual was labeled as an IHD case based on having a primary or secondary diagnosis of any of a list of ICD-10 codes and on self-reported questionnaire data (Additional file 2: Table S2). We defined MI as a subset of IHD, with a more restrictive set of ICD-10 codes (Additional file 2: Table S3). Individuals with MI who had their first MI event by the age of 50 were labeled as early MI cases. We used time stamps on HES data for the MI ICD-10 codes to find the age of an individual’s first MI event and excluded questionnaire data due to lack of information about diagnosis date. The prevalence and cohort statistics and for each condition in the European-only population are described in Table 1.

**Table 1:** Cohort data for IHD, MI, and early MI, and European UK Biobank Population

	Ischemic heart disease (IHD) (8.96% prevalence, 39,516 total cases)	Myocardial infarction (MI) (3.61% prevalence, 15,930 total cases)	Early MI (0.15% prevalence, 669 total cases)	UK Biobank European Subset (441,101 individuals)
**Sex**	33% female (12,890) 67% male (26,626)	23% female (3599) 77% male (12,331)	14% female (96) 86% male (576)	54% female (238,358) 46% male (202,743)
**Smoking status**	13% current (5199) 47% past (18,387) 40% never (15,930)	16% current (2564) 49% past (7774) 35% never (5592)	28% current (191) 43% past (285) 29% never (193)	10% current (44,710) 35% past (155,055) 55% never (241,336)
**Diabetes type 2**	19% (7480)	20% (3214)	26% (177)	5% (23,634)
**Family history heart disease**	45% (17,970)	48% (7599)	58% (386)	24% (103,639)
**Mean systolic blood pressure**	143.5 ± 20.08 mmHg	142.1 ± 20.5 mmHg	133.7 ± 18.6 mmHg	140.1 ± 19.6 mmHg
**Mean BMI**	29.1 ± 5.0 kg/m^2^	29.0 ± 4.8 kg/m^2^	30.3 ± 5.5 kg/m^2^	27.4 ± 4.8 kg/m^2^
**Mean birth year**	1947 ± 6 years	1947 ± 6 years	1957 ± 6 years	1951 ± 8 years
**Mean Cholesterol**	5.2 ± 1.3 mM/L	5.0 ± 1.3 mM/L	4.7 ± 1.2 mM/L	5.7 ± 1.1 mM/L
**Mean triglycerides**	2.0 ± 1.1 mM/L	2.0 ± 1.1 mM/L	2.2 ± 1.3 mM/L	1.8 ± 1.0 mM/L
**Mean LDL**	3.2 ± 0.9 mM/L	3.1 ± 0.9 mM/L	2.9 ± 0.9 mM/L	3.6 ± 0.9 mM/L

All data displayed in this table is for individuals of the European-only subset in the UK Biobank. Entries in brackets indicate number of cases or individuals

### Cross-validation overview

For each risk prediction model, we embedded the logistic regression (LR) algorithm in a 10-fold cross-validation framework (Fig. 1b). The outcome variable was one of the three labels (IHD, MI, or early MI), which was modeled as a function of a subset of features from the 14 available. The total samples were randomly split into 10 non-overlapping groups, and for each fold of the cross-validation, each group (10% of the data) was iteratively designated as the test set, while the remaining nine groups (90% of the data) were set aside as the training set. In order to avoid bias, the LR algorithm was trained using a balanced subset of this training set [37, 38], which consisted of all the cases in the training set and an equal-sized random set of controls from the training set (Additional file 2: Table S4). The test set was unbalanced in order to assess the performance of the trained algorithm on a completely random subset of the population.

### Statistical analysis

During each fold of cross-validation, each individual was binned into a percentile and the proportion of cases in each bin was calculated. Data about the area under the curve (AUC) and the weights of the features were also saved. After a given model was trained using ten folds of cross-validation, the data saved from each fold was used to calculate 95% confidence intervals for the proportion of cases (prevalence) in each percentile bin, the AUC, and feature weights, based on the t-distribution of ten trials. The resulting trained models represent IHD, MI, and early MI risk scores and were used to assess disease risk. In this study, prevalence is considered to be the proportion of cases in a subset of the population and represents the probability that a given individual is a case for the condition of interest. Relative risk is defined here as the probability that an individual is a case given that he or she is classified as being at or above a given percentile threshold of the risk score (e.g., 99th percentile, 90th percentile) over the probability that the individual is a case given that he or she is classified as being below that percentile threshold. The ratios between the prevalence of each condition at different percentiles of the risk score, and between the prevalence of each condition at a given percentile of the risk score and the overall population prevalence, were also measured as proxies for relative risk. The AUC resulting from each trained model was used as the primary measure of risk discrimination for each score.

### Propensity matching

In order to assess whether risk assessment differs among individuals as a function of their medication usage, we re-evaluated overall genetic and modified risk on propensity matched samples. Individuals were classified based on self-reported medication intake, by dividing the sample into three groups of sizes indicated in Additional file 2: Table S5, based on data from UKB data field 20,003 (“Treatment/medication code”)—those taking any of a list (see Additional file 2: Table S6) of blood pressure medications and not any statins (“BP Meds Only”), those taking any of a list of statins but not blood pressure medications (“Statins Only”: Additional file 2: Table S7), and those not taking any blood pressure medications (“Neither”).

Propensity matching of individuals within each subset was performed by first training a standard logistic regression algorithm on the full dataset of all UKB individuals using the selection of features the individuals were to be matched for, namely the 9 non-fixed features. We then set aside the sample subset which contained the smallest number of individuals and trained a k-nearest neighbors algorithm (k-NN) on each of the remaining subsets using the decision function scores that resulted from the logistic regression algorithm and fitted the model to the smallest sample subset. This resulted in a set of individuals in each of the larger sample subsets that was matched with the individuals in the smallest sample subset based on having similar characteristics for the selected features. The prevalence for IHD, MI, and early MI for each decile were then compared between the resulting matched subpopulations. This process resulted in matched subpopulations for self-reported medication intake. Owing to the reduced number of individuals on medication, we only computed the prevalence for each decile of risk, which was done for the full model with 18 PRS + established-at-birth + non-fixed features.

## Results

Here, we describe (a) to what extent PRS improve prediction when included with established-at-birth as well as non-fixed conventional risk factors, (b) how PRS developed for general IHD also predict early onset MI, (c) an adjustment for ancestry that facilitates risk assessment in a mixed ancestry population, (d) evaluation of which non-fixed risk factors correlate with genetic risk, and (e) differences in risk profiles between propensity-matched individuals taking statins, blood pressure medications, or neither.

Each of the four PRS (FDR202, GRS46K, 1.7M, and 6M) individually achieves similar accuracy in discriminating cases of IHD, MI, or early MI (Additional file 2: Table S8). Combining all four PRS together leads to better performance than any of the individual PRS. Note that a metaGRS score [22], which is a weighted sum of FDR202, GRS46K and 1.7M, also performs better than each individual score, but did not add to the combined prediction in our logistic regression models. When the results for both the European-only cohort and the larger UK Biobank cohort with individuals of all ancestries were compared, it was found that the results for all experiments were nearly the same between the two groups, within the standard error of each other. To avoid repetition, the results shown hereafter are those for the European-only cohort unless otherwise noted.

### Joint modeling of genetic and clinical factors implies limited incremental value of PRS

The first step in this analysis was to combine the four PRS with established-at-birth risk factors—biological sex and ancestry—as features in the logistic regression algorithm. Before doing so, we trained two univariate logistic regression models using just sex and ancestry as features. Sex alone provides approximately 3-fold discrimination of MI risk, reflecting the observed prevalence of 7% in men and 2.3% in women enrolled in the UKB population. Residual ancestry captured by the first four genotypic PC in the European ancestry British subset had little effect. A model combining all four PRS performs similarly to the 6M SNP PRS alone, with a modal risk of 3.6% and the risk for those individuals above the 97th percentile at least 3-fold greater than this (Table 2; red curve in Additional file 1: Fig. S2). When these PRS are combined with sex and ancestry features, the top 15% of the total sample is at more than 3-fold higher risk than the average, and the top percentile has a prevalence approximately 17%. This alone is a notable improvement over the PRS-only scores.

**Table 2:** Percentile vs. prevalence statistics for multivariate logistic regression

	Percentile	PRS only (4-feature model)	Established-at-birth only (5-feature model)	Established-at-birth plus PRS (9-feature model)	Established-at-birth plus non-fixed (14-feature model)	Established-at-birth plus non-fixed plus PRS (18-feature model)
**IHD**	**Top percentile**	21.95% ± 1.39	13.52% ± 1.10	29.98% ± 1.65	55.71% ± 1.83	60.25% ± 1.37
**IHD**	**Middle percentile**	8.76% ± 0.80	5.48% ± 0.63	7.69% ± 0.89	5.34% ± 0.70	4.50% ± 0.69
**IHD**	**Lowest percentile**	3.45% ± 0.47	4.56% ± 0.71	2.79% ± 0.63	0.48% ± 0.18	0.41% ± 0.12
**MI**	**Top percentile**	11.38% ± 1.05	6.60% ± 0.72	16.78% ± 1.10	30.95% ± 1.63	35.90% ± 1.66
**MI**	**Middle percentile**	3.19% ± 0.35	1.72% ± 0.30	1.95% ± 0.42	1.54% ± 0.46	1.11% ± 0.20
**MI**	**Lowest percentile**	0.68% ± 0.31	1.98% ± 0.34	0.59% ± 0.22	0.09% ± 0.09	0.14% ± 0.14
**Early MI**	**Top percentile**	0.88% ± 0.32	0.27% ± 0.28	1.34% ± 0.42	4.15% ± 0.52	4.67% ± 0.83
**Early MI**	**Middle percentile**	0.07% ± 0.08	0.02% ± 0.05	0.07% ± 0.11	0.02% ± 0.05%	0.00% ± 0.05
**Early MI**	**Lowest percentile**	0.00% ± 0.00	0.02% ± 0.05	0.00% ± 0.00	0.00% ± 0.00	0.00% ± 0.00

Entries are observed prevalence of IHD, MI, or early MI, plus or minus 95% CI from 10-fold cross-validation in each indicated model for the top, middle, and bottom 1% of polygenic risk

**Fig. 2:**
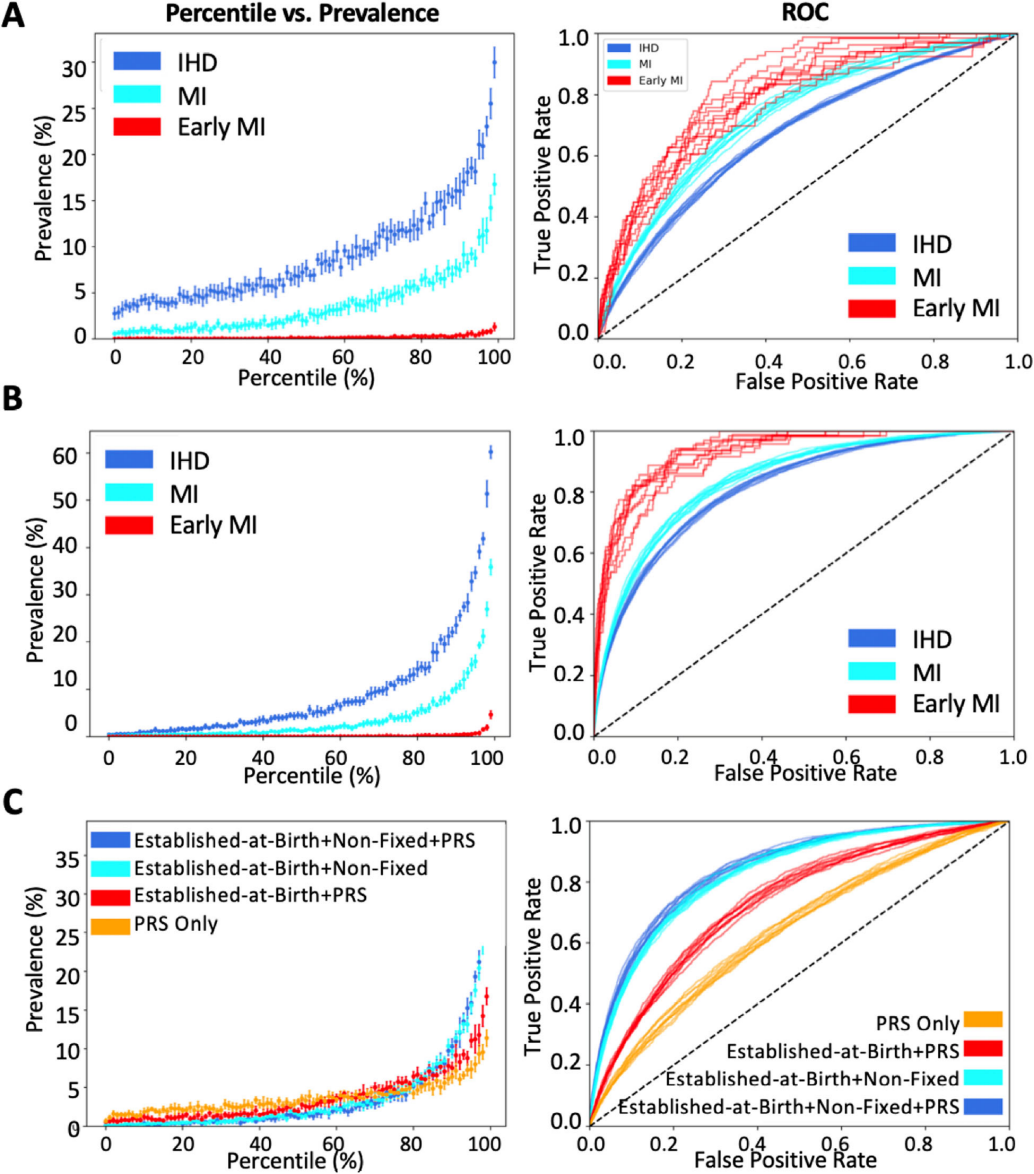
Modeling cardiovascular risk in the UKB with genetics and non-fixed factors. Panels on the left-hand side show the prevalence in the White British and Irish European UKB sample versus modeled risk percentile, for the 9% with ischemic heart disease (IHD, dark blue), approaching 5% who have had a myocardial infarction (MI, cyan), and 0.15% who had MI before the age of 50 (early MI, red). Panels on the right show receiver-operating curves of sensitivity against specificity. Each curve represents one of 10 cross-validated estimates, with point estimates plus standard deviation on the left. **a** 9-feature established-at-birth model. **b** 18-feature full-model. **c** Effect of adding features for MI prediction alone, yielding 4-feature, 9-feature, 14-feature, and 18-feature models as outlined in Fig. 1a

Subsequently, we added non-fixed risk factors to the logistic regression models. Figure 2a contrasts the percentile vs. prevalence curves for IHD, MI, and early MI for the 9-feature (PRS, sex, and ancestry) scores. In this and related figures, in the left-hand panels, the *x*-axis represents the percentile rank for the model, and the *y*-axis represents the prevalence, which is equivalent to the positive predictive value for individuals in that percentile. Figure 2b contrasts percentile vs. prevalence curves for the 18-feature full models for IHD, MI and early MI, and Fig. 2c shows how accuracy increases as features are added, for MI. The right-hand panels are the corresponding receiver operating characteristic (ROC) curves, namely sensitivity against 1-minus-specificity, the area under the curve (AUC) of which provides a guide to the overall accuracy of the model. Similar trends are shown for IHD (Additional file 1: Fig. S3). Salient results are also reported in Table 2 (prevalence by percentile for each model) and Additional file 2: Table S9 (AUCs).

The combination of genetics and non-fixed risk markedly improves prediction over genetics alone. For MI, the prevalence at the top percentile of risk increases from 11% in the genetics only model to 36% in the combined model, and equally notably the prevalence in the bottom percentile reduces from 0.7 to 0.1%. The AUC increases from 0.64 to 0.84. Similar improvements are seen for IHD and early MI, discussed further below.

As importantly, the contribution of the genetic risk assessment does not simply add to the non-fixed factors in the full model and is in fact notably limited. It is greatest in the top two percentiles of risk, rising for example from 30.6% ± 1.2 prevalence of MI to 35.6% ± 1.9 for the top percentile, which is an approximately 10% relative increase. However, for the remainder of the percentiles in Fig. 2c, the percentile vs. prevalence points representing the full model and clinical model without PRS are essentially overlaid, and the ROC curves are barely distinguishable. Table 3 shows the weightings for each factor, with cholesterol, sex, and age (birth year) making large contributions and the 6M SNP PRS accounting for most of the genetic contribution. Additional file 2: Table S10 shows the weightings for the 9-feature established-at-birth model including PRS.

**Table 3:** Feature weights resulting from multivariate logistic regression

Feature	Weight (IHD Score)	Weight (MI Score)	Weight (early MI Score)
**FDR202**	0.09 ± 0.01	0.14 ± 0.00	0.24 ± 0.01
**GRS46K**	0.16 ± 0.00	0.23 ± 0.00	0.24 ± 0.02
**1.7M**	0.12 ± 0.00	0.17 ± 0.00	0.44 ± 0.01
**6M**	1.83 ± 0.02	1.85 ± 0.02	0.23 ± 0.01
**Diabetes type 2**	1.00 ± 0.01	0.80 ± 0.01	1.10 ± 0.05
**Family History of Heart Disease**	1.01 ± 0.00	0.98 ± 0.01	1.54 ± 0.04
**Sex**	0.65 ± 0.00	1.02 ± 0.01	1.46 ± 0.04
**Birth year**	− 0.78 ± 0.00	− 0.79 ± 0.00	1.22 ± 0.02
**Systolic blood pressure**	− 0.03 ± 0.00	− 0.07 ± 0.00	− 0.37 ± 0.01
**BMI**	0.23 ± 0.00	0.19 ± 0.00	0.36 ± 0.02
**Cholesterol**	− 0.34 ± 0.01	− 0.39 ± 0.00	− 0.84 ± 0.02
**Triglycerides**	0.18 ± 0.00	0.19 ± 0.00	0.32 ± 0.01
**LDL**	0.17 ± 0.01	0.27 ± 0.02	− 0.77 ± 0.07
**Smoking status**	0.34 ± 0.00	0.45 ± 0.00	0.57 ± 0.02
**PC1**	0.17 ± 0.00	0.05 ± 0.04	− 0.09 ± 0.48
**PC2**	0.16 ± 0.02	0.16 ± 0.04	− 0.39 ± 0.30
**PC3**	0.01 ± 0.00	0.02 ± 0.00	0.05 ± 0.01
**PC4**	0.00 ± 0.00	0.01 ± 0.00	0.02 ± 0.01

Feature weights from training the 14-feature logistic regression algorithm, with 95% confidence intervals from 10-fold cross-validation

### PRS predicts early onset MI

**Fig. 3:**
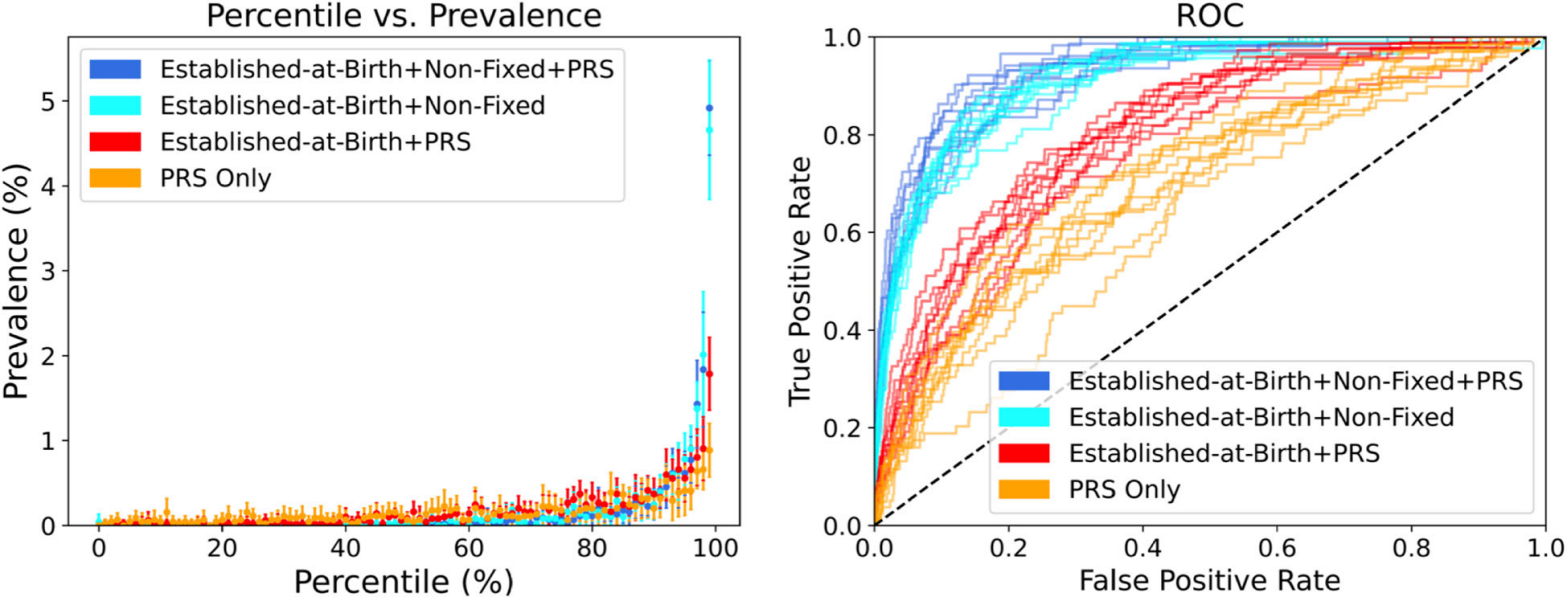
Prevalence vs. risk percentile and ROC plots for four risk models for early MI. Plots show the predictive> performance and accuracy for the polygenic risk scores (PRS) only model (yellow), the 9-feature established-at-birth plus PRS model (red), 14-feature established-at-birth plus non-fixed model without PRS (light blue), and the 18-feature full model (dark blue). Each curve represents one of 10 cross-validated estimates, with point estimates plus standard deviation on the left

Despite the genetic risk scores being developed to predict coronary events at any age, and an overall prevalence of MI before the age of 50 of just 0.15%, the PRS models provide significant discrimination of high-risk individuals for early MI. There is a more than 10-fold elevated prevalence of early MI in the top percentile of the 9-feature PRS, sex and ancestry score relative to the sample mean (almost 1.8% relative to 0.17%), and an optimal score would identify 50% of cases with a less than 20% false positive rate (Fig. 3 ROC panel, red curve). This is a very marked improvement over the established-at-birth factors alone (Table 2) and is in agreement with the findings of Mars et al. [26] in FinnRisk that net reclassification of cardiovascular disease is improved for early onset cases using a similar PRS. Nevertheless, both the 14-feature clinical model without PRS and the full 18 feature model with PRS are both remarkably predictive in the top percentile, each approximately 5%, but genetics once again adds little additional discrimination. These results imply that non-fixed risk factors already impact risk of early onset MI. Interestingly, the logistic regression weights of the 1.7M, GRS46K, and FDR202 scores are larger than that of the 6M SNP PRS for the early MI PRS-only and 7-feature models, in contra-distinction to the IHD and MI models.

### Adjusting for ancestry by logistic regression

**Fig. 4:**
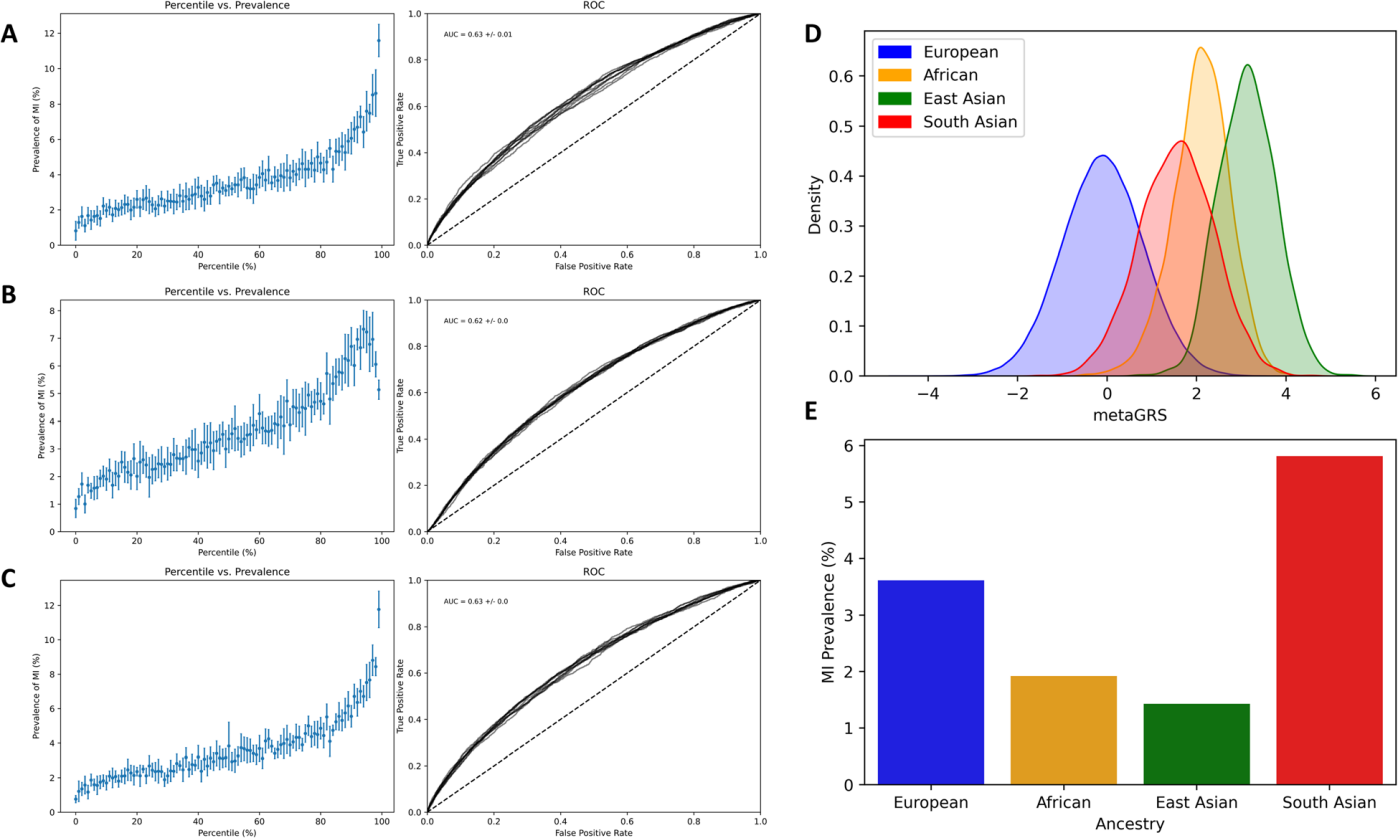
Adjustment for ancestry by logistic regression. **a**-**c** Prevalence vs. Risk percentile plots for MI for the White British and Irish Europeans (**a**), full UK Biobank (**b**) and a model including genotypic PC1 and PC2 to control for ancestry (**c**). **d** shows frequency distributions of European-derived PRS in each of the indicated ancestry groups, standardized to equivalent size (European *n* = 441,173; African 7190; East Asian 1471; South Asian 7413). **e** Prevalence of MI in each ancestry group

As shown by others [39–41], the frequency distribution of polygenic risk scores derived with weights observed in one ancestry group are generally shifted in other ancestry groups due to cumulative subtle differences in allele frequencies. For example, the metaGRS derived by Inouye et al. [22] is higher by more than a standard deviation unit in each of the African-, East-Asian-, and South-Asian ancestry groups in the UKB (Fig. 4d). Since prevalence of MI is actually lower in the African and East Asians (Fig. 4e), the raw metaGRS actually predicts reduced prevalence in the highest risk percentiles for the full UKB (Fig. 4b).

We adjusted for this bias by including the first four principal components of the genotype matrix in the 9-feature logistic regression model with sex and the four PRS. The resultant prevalence vs. risk percentile curves with 10-fold cross-validation are essentially indistinguishable from those of the White British and Irish European only sample (Fig. 4c compared to Fig. 4a). This adjustment similarly controls for ancestry in the models including non-fixed factors. Though an apparent improvement in relative risk prediction over failing to account for ancestry, we caution that there is no guarantee that rank order of risk is preserved in other ancestry groups and note that the sample sizes are too small to evaluate prediction in each population. Transfer of the risk scores derived here to other ancestries is thus not recommended, though we do show that the simple adjustment accounts for some of the bias.

### Non-fixed factors influencing genetic risk effects at birth and middle age

**Fig. 5:**
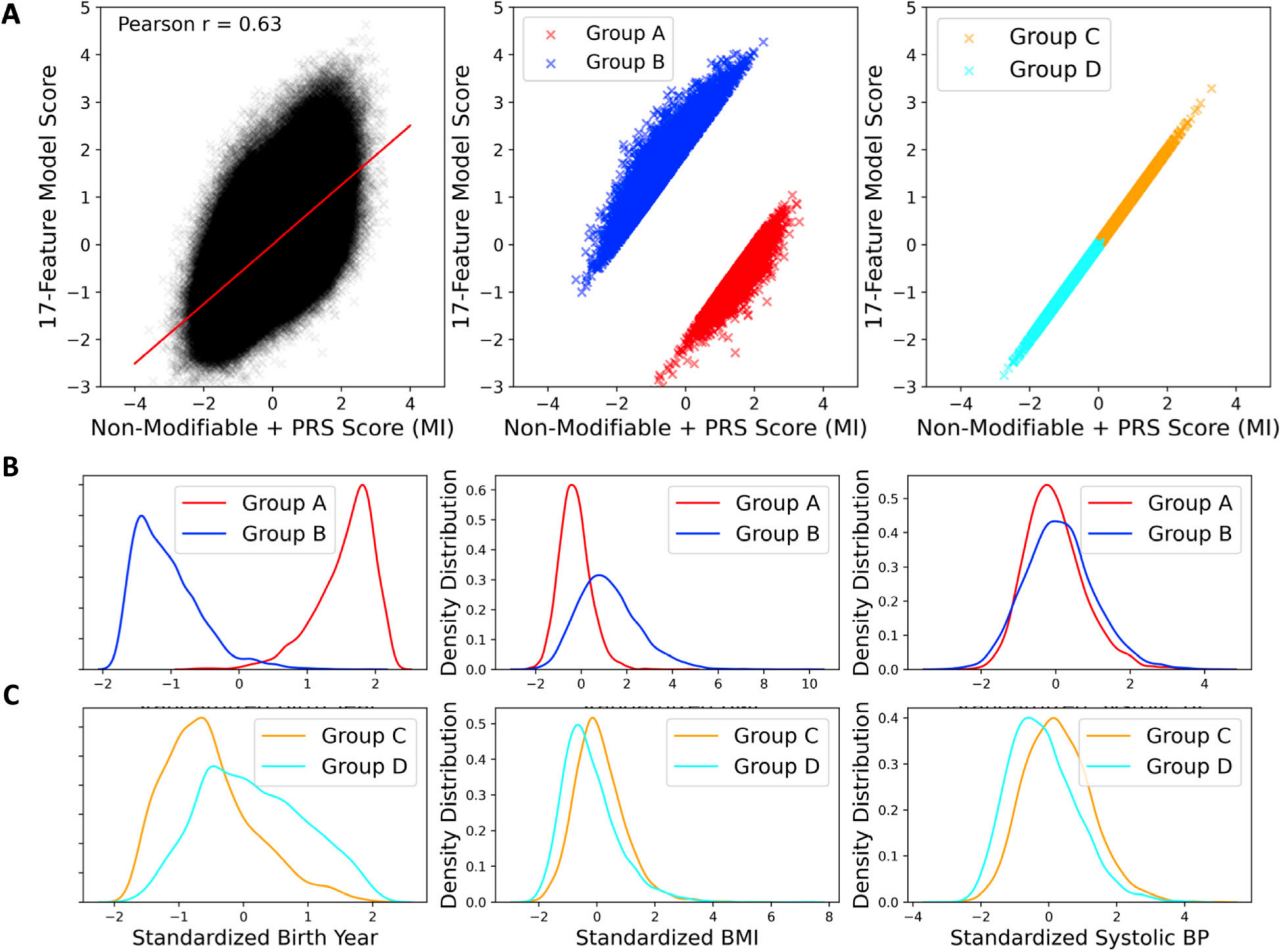
Comparison of MI Risk Scores with and without non-fixed risk factors. **a** Scatterplots of the full model (18-feature established-at-birth + non-fixed + PRS score) against the non-modifiable risk model (9-feature established-at-birth + PRS score). Individuals below the regression line (red, Group A in the middle panel) have comparatively lower predicted risk later in life compared to their predicted risk at birth, whereas those above the regression line (blue, Group B) have higher predicted risk of MI later in life; both groups represent the 2% extremes. The right -hand panel highlights individuals with relatively low risk according to both models (Group C, orange) or relatively high risk according to both models (cyan, Group D). **b**, **c** Standardized distributions of indicated non-fixed risk factors for the selected subsets of individuals, implying that high risk individuals are older and more overweight

In order to compare the risk assessments of individuals “at birth” and “in middle age” after known clinical risk factors such as BMI, smoking, lipids, and blood pressure have had time to exert their influence, we contrasted the 9-feature established-at-birth plus PRS score with the 18-feature full model score. Figure 5 shows scatterplots, where we have also selected four groups of individuals for additional assessment. The all-features score also incorporates family history, and age, recognizing that the cumulative probability of MI increases over the age of 50. The overall Pearson correlation is *r* = 0.63, implying that the scores are correlated, but that clinical risk is modified by both genetics and lifestyle. Those individuals in the two 98th percentile tails for the residual variance for the regression between the scores at birth and in middle age were identified and then separated into two groups, where group A have less-than-expected risk in middle age relative to their genetic risk at birth, while group B have greater-than-expected risk. Those individuals at or below the 2nd percentile of residual variance between their scores at birth and in middle age were also identified, and then separated into two groups of higher (group C) or lower (group D) overall risk by both models.

The three possible contributing continuous-trait explanations for these group differences are considered in Fig. 5b,c. Individuals in group A tend to be younger and have a slightly lower BMI than those in group B, consistent with the expectations that aging and excess weight gain increase the odds of having an MI. Interestingly, these two groups do not seem to differ substantially with respect to systolic blood pressure. The four possible categorical explanations are shown in Additional file 1: Fig. S4. Absence of diabetes, never smoking, and not having a family history of coronary disease all associate with reduced mid-life risk compared to baseline genetics. The influence of family history may suggest either a genetic predisposition that is not captured by the polygenic risk assessment or possibly lifestyle or socioeconomic predisposition associated with upbringing and family influences. These results provide direct evidence that genetic risk factors can to some extent be offset or over-ridden by lifestyle choices and practices that influence the known clinical risk factors.

The data for the analysis of groups C (high risk at birth and in middle age) and D (low risk at birth and in middle age) are also shown in the figures. Those in group C tend to be older and have higher BMI as well as systolic blood pressure. High lipids or type 2 diabetes (~ 4% and ~ 6%, respectively) are not strongly over-represented in this group, but clear differences in smoking status and family history do discriminate groups C and D.

### Propensity matching risk as a function of medication regimen

**Fig. 6:**
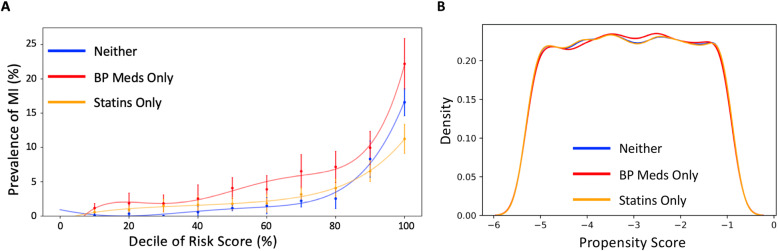
Effect of genetics on prevalence as a function of medication. **a** Prevalence vs. risk decile plots showing overall greater apparent influence of genetics for patients on blood pressure medication. This analysis is based on the 18-feature logistic regression model run for White British and Irish-only patients with MI as the label. **b** Frequency distributions of propensity scores showing close similarity for the three groups

Contrasting Figs. 3 and 5 of Inouye et al. [22], it is apparent that cumulative risk of incident CAD for those taking lipid-lowering drugs and/or anti-hypertensive medicines is elevated as much as 50% for both men and women across all levels of genetic risk. This is surprising given the documented effectiveness of these interventions and may reflect differences in clinical features of those who take them. To examine the impact of medications in the context of genetic risk, we performed a propensity matching analysis, identifying all cases and controls on just blood pressure medications, on just statins (see Additional file 2: Tables S5, S6, S7), or not taking medication for cardiovascular prevention. Equal sized mutually exclusive groups were identified and matched for all 18 genetic and clinical features. We then recomputed the multivariable logistic regression models and evaluated deciles rather than percentiles of risk, owing to the reduced sample size of the test datasets. Figure 6 shows that the statin-only and no-medication groups have similar prevalence vs. risk profiles, whereas the blood pressure medication group show higher prevalence for all individuals in the top half of the risk profile. Regardless of medication status, there is approximately 10-fold higher prevalence in the top decile of risk than the median. To assess the extent to which adding medications data as features to the standard logistic regression models described in the previous section, we additionally ran a 20-feature model that included binary features for medication intake. This model is described in Additional file 1: Fig. S5 and in Additional file 2: Tables S11, S12, and S13 and follows the same overall trend as the 18-feature model but actually has a slightly higher AUC and prevalence at the top percentiles of risk.

## Discussion

Considerable interest in the clinical use of PRS to identify individuals at high risk of coronary artery disease has been generated by the recent revelation that many more people are predisposed to cardiovascular disease due to their genetic background from common variants than due to rare variants of large effect. Classical risk factors such as hyperlipidemia do not individually generate such discrimination, but we show here that smoking, elevated BMI, hyperlipidemia, hypertension, and family history when collectively combined with age generate a retrospective diagnostic score that is highly accurate for prevalent MI and IHD in the UK Biobank. In this context, polygenic risk assessment adds very little utility.

A likely reason is that the PRS are indirectly associated with some or all of the risk factors. Additional file 1: Fig. S6 indicates that this is indeed the case, since the correlation between the combined PRS and all but smoking status ranges between 0.05 and 0.10, being highest for lipid status in males. Though these correlations are low, this is because known genetics only explains a fraction of each non-fixed risk factor. Nevertheless, if the genetic risk for IHD is mediated through these factors, then once individuals reach late middle age, when MI events start to become more prevalent, then the genes have already exerted their influence, and the PRS would not be expected to add appreciably to risk prediction.

A corollary of this would be that early ascertainment of elevated risk ought to encourage individuals to pursue lifestyle or medical interventions to prevent the onset of sub-clinical disease. Consistent with this notion, the four PRS also discriminate individuals who experienced an MI before the age of 50. Even excluding the top 5% of individuals with respect to combined clinical risk, genetic risk alone provides more than fivefold elevated risk for one quarter of the sample. The observation that the PRS derived by Inouye and collaborators make a greater contribution to the models than the 6M SNP score derived by [19], which inverts the situation for total MI, suggests that genetic risk for early onset MI remains to be well defined. GWAS based on a very large sample of early onset cases will be needed to establish the genetic correlation between early and late onset coronary artery disease and to refine the PRS.

Importantly, the results for all experiments in the European-only cohort mirrored those of the full UK Biobank cohort, possibly suggesting that the findings of this paper apply broadly across ancestries. However, it is worth noting that the European-only cohort comprises over 90% of the full UK Biobank cohort in this study. Thus, after adjustment of the logistic regression models for ancestry, individuals of non-European ancestries may not have comprised a large-enough proportion to impact the overall prediction accuracy. In general, it is thought that PRS need to be specific to each ancestry in order to optimize the amount of variance explained and hence accuracy [40–42]. The issue of how to adjust for admixture in mixed ancestry individuals remains to be addressed, possibly including local as well as global (PC) estimates in the modeling.

The results of the propensity-matched medication analyses were surprising in two regards. First, since it is established that statin usage generates significantly greater reduction in prevalence of MI in high PRS strata [43], a flattening and overall reduction of the prevalence vs. percentile score might have been expected. Instead, the statin curve overlays closely on that for individuals not taking medications. If the statins were prescribed prior to initial measurement of the clinical factors that were used in the computations, then they may have already reduced risk in those individuals who propensity match with non-medicated individuals. Second, the curve for blood pressure medication lies significantly above the other two from the 5th to the 10th deciles, indicating that these individuals remain at higher risk than their matched peers. This implies prima facie that the genetics captured by the PRS has a greater impact for individuals on blood pressure medication than those on statins [44] and, alternatively, that statins effectively blunt the impact of the genetics, whereas the blood pressure medications do not, and hence that the PRS does not efficiently capture some aspect of risk that is independent of lipids and BMI but is correlated with hypertension. Possibilities include inflammation and endothelial cell function. Incorporation of PRS for biomarkers of these and other cardiac endophenotypes may improve the accuracy of genetic prediction of MI even further.

### Limitations

In this study, we assessed the additive value of PRS in IHD, MI, and early MI risk prediction when included in risk assessment along with conventional risk factors, but there were several limitations. First, because the study did not assess longitudinal 10-year risk but rather assessed risk based on risk factor burden at a given age, the 14- and 18-feature scores that include non-fixed risk factors are not directly comparable to time-to-event risk scores, such as the Framingham Risk Score, which are most commonly used in the literature [4]. However, given that scores that provide stable risk estimates based on risk factor burden at a given age are currently being recommended for clinical use by Canadian Heart Association guidelines [9], these scores can complement time-to-event (for example 10-year) risk scores. Furthermore, we note that our results are in good agreement with those based on incidence in the UKB reported by others. The AUCs for IHD reported for PRS alone by [22, 24] and here are 0.61, 0.62, and 0.61 respectively, for conventional risk factors are 0.76, 0.67, and 0.80, and for the combined models 0.78, 0.70, and 0.81, in each case implying just a modest increment due to PRS. It is known that prevalent versus incident comparisons tend to inflate model performance, but the suspicion that Elliott et al.’s modeling [24] improves on that of Inouye et al. [22] because the former used quantitative rather than categorical measures of hyperlipidemia is not confirmed by our analysis because slightly higher accuracy was attained with the categorical data as shown in Additional file 2: Table S14.

A second limitation was due to the quality and availability of UKB data. For example, lifestyle or clinical risk factor data from early life or young adulthood was not available, given that the UKB population was mostly between the ages of 40 and 69 at the time of recruitment [27]. Thus, the 14- and 18-feature early MI scores classify individuals as cases or controls based on their risk factor burden in middle age rather than earlier in life. Additionally, limiting the analysis only to incident cases reduces the sample size substantially, since many individuals in the analysis were diagnosed as cases prior to recruitment into the UK Biobank. In fact, 73% of the MI cases and 49% of the IHD report or can be inferred to have been diagnosed prior to enrolment in the study (Additional file 2: Fig. S7 which shows the distribution of times to incidence for both diagnoses). Furthermore, the timing of onset of certain risk factors as compared to diagnosis of disease is not clear, and it could be that some patients were diagnosed with IHD or experienced an MI prior to the elevation of their BMI or lipids. This may have resulted in inflated performance of the clinical risk factors in the scores. We refer readers to the discussion in [24] of the impact of model calibration on their analysis of incident risk.

Another limitation is that the LR algorithm was trained and tested on the same dataset. Although we used a tenfold cross-validation approach, which has been shown to be better than splitting the dataset into a training set and just one validation set, an even better approach would be to externally validate the study one or more independent datasets. It should also be noted that some of the PRS included here were partially trained on UK Biobank data, which will also inflate their performance.

Finally, the PRS analysis is also limited by the quality of the available UKB genotype data which relies on imputation for many of the markers. Moreover, it has been established [45] that the UKB should not be assumed to be an accurate representation of the British population in general, due to ascertainment biases in the location of recruitment centers and nature of voluntary participation. Although the 6M SNPs score was published as recently as 2018, and had the highest weighting compared to the other PRS scores in most analyses, it is to be expected that even more accurate PRS will be developed that may further elucidate the role that genetics plays in heart disease risk prediction.

## Conclusions

The results of this study confirm and expand upon recent studies, collectively establishing that elevated risk of cardiovascular disease in middle age is mostly influenced by clinical and lifestyle factors rather than by independent genetic susceptibility. However, polygenic risk scores may nevertheless be useful for predicting risk at birth, when other risk factors are unknown, helping to identify individuals most susceptible to development of heart disease.

## Supplementary Information

**Additional file 1: Supplementary Figures** **Figure S1.** PC1 and PC2 as Proxies for Ancestry. **Figure S2.** Prevalence vs. Risk Percentile plots and ROC curves for MI. **Figure S3.** Prevalence vs. Risk Percentile plots and ROC curves for IHD. **Figure S4.** Distribution of categorical risk factors by non-fixed risk group. **Figure S5.** Prevalence vs. Risk Percentile plots and ROC curves for models including medication use. **Figure S6.** Correlations between PRS only scores and non-fixed features. **Figure S7.** Time between enrolment and diagnosis in the UK Biobank.

**Additional file 2: Supplementary Tables**. **Table S1.** Description of the features used to train logistic regression algorithm. **Table S2.** Classification of ischemic heart disease (IHD) cases. **Table S3.** Classification of myocardial infarction (MI) cases. **Table S4.** Sample sizes for cross-validation algorithm for European-only analyses. **Table S5.** Total number of individuals in each category of medication intake. **Table S6.** List of UKB field 20,003 medication codes used for “Blood Pressure Medication - Any” classification for propensity matching analysis. **Table S7.** List of UKB field 20,003 medication codes used for “Cholesterol Medication - Statins” classification for propensity matching analysis. **Table S8.** AUCs for univariate logistic regression with individual compared to joint PRS. **Table S9.** AUCs for multivariate logistic regression with different feature combinations. **Table S10.** Feature weights from the Established-at-Birth plus PRS (9-Feature) models. **Table S11.** Feature descriptions for three additional features included in the 20-Feature models for IHD, MI, Early MI. **Table S12.** Percentile vs. prevalence statistics for multivariate logistic regression for 20-feature model. **Table S13.** Feature weights from the 20-Feature models for IHD, MI, Early MI. **Table S14.** AUCs for multivariate logistic regression with different feature combinations, but using categorical hyperlipidemia in place of cholesterol, TG, and LDL measurements.

## Data Availability

The original datasets used to conduct the study are available from the UK Biobank Resource, https://www.ukbiobank.ac.uk/, under approved license 17984 to GG. The associated files contain all of the data that is needed to replicate the findings of this study.

## Abbreviations

AUC: Area under the receiver operating (sensitivity-specificity) curve
CAD: Coronary artery disease
GWAS: Genome-wide association study
IHD: Ischemic heart disease
MetaGRS: A weighted sum of three PRS for CAD
MI: Myocardial infarction
PRS: Polygenic risk score
UKB: UK Biobank study
