# Supplementary material for: Highly elevated polygenic risk scores are better predictors of myocardial infarction risk early in life than later
Source: Genome Med. 2021 Jan 28;13:13. doi: 10.1186/s13073-021-00828-8 (PMC7845089; doi:10.1186/s13073-021-00828-8)
Supplement: Supplementary file 1 — Additional file 1: Supplementary Figures Figure S1. PC1 and PC2 as Proxies for Ancestry. Figure S2. Prevalence vs. Risk Percentile plots and ROC curves for MI. Figure S3. Prevalence vs. Risk Percentile plots and ROC curves for IHD. Figure S4. Distribution of categorical risk factors by non-fixed risk group. Figure S5. Prevalence vs. Risk Percentile plots and ROC curves for models including medication use. Figure S6. Correlations between PRS only scores and non-fixed features. Figure S7. Time between enrolment and diagnosis in the UK Biobank. [file 13073_2021_828_MOESM1_ESM.docx]

**ADDITIONAL FILE 1: SUPPLEMENTARY FIGURES**


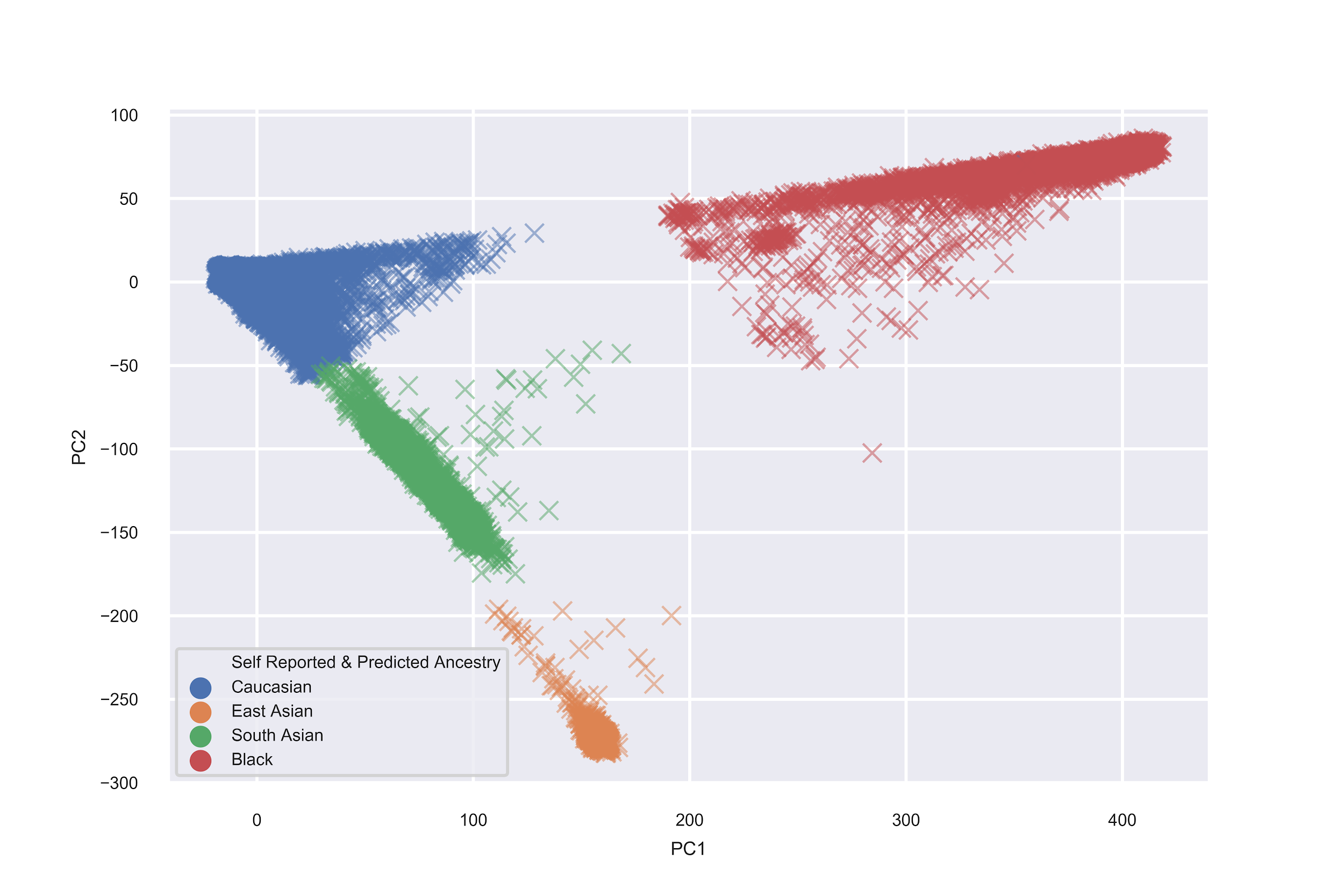


**Figure S1. PC1 and PC2 as Proxies for Ancestry**. Genotype PC1 and PC2 coordinates for individuals in the UK Biobank colored by k-means clusters with k=4, showing only individuals who also self-report membership in one of the four indicated ancestry groups. The Blue group are the White British and Irish. Residual genetic variation was accounted for by including both PCs as established-at-birth covariates in subsequent analyses.

**
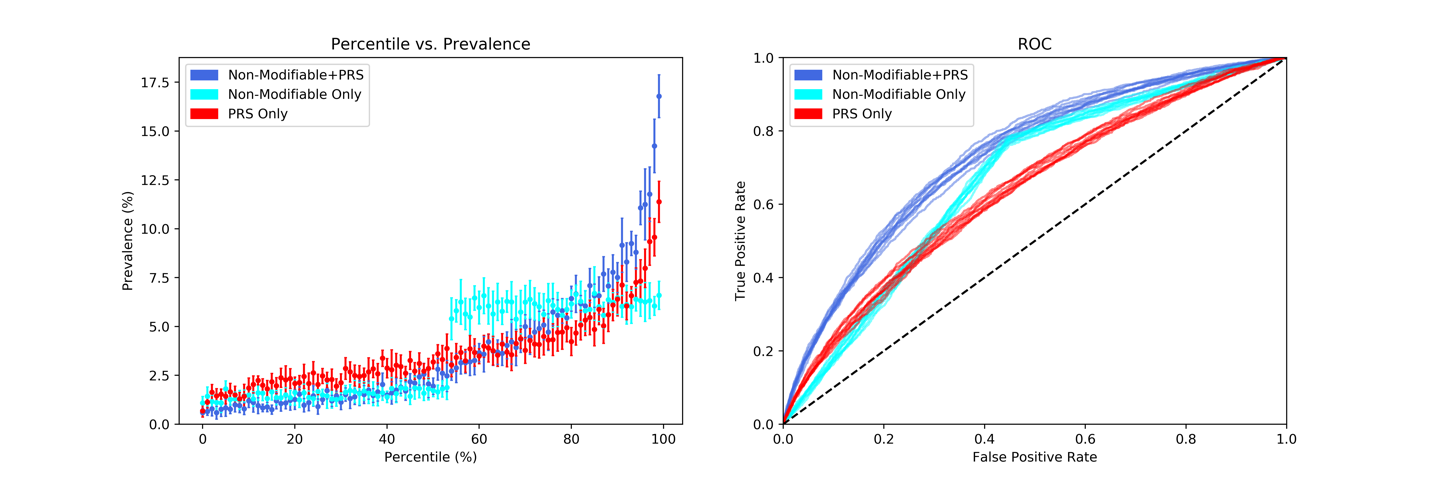
**

**Figure S2. Prevalence vs. Risk Percentile plots and ROC curves for MI.**  Similar to Figures 1-3 in the main manuscript, these panels plot the prevalence of cases (MI) in each percentile of risk score, as well as the receiver operating curve of sensitivity against (1 minus specificity). The light blue established-at-birth only score includes Sex and the first four genotypic PC, and mostly discriminates higher prevalence of MI in males. The red PRS only score includes the four polygenic risk scores with 202, 46K, 1.7M and 6M variants. The dark blue curve combines both of these sets of variables, identifying approximately 20% of the population with more than 10% risk of MI to date in the UKB study.

**
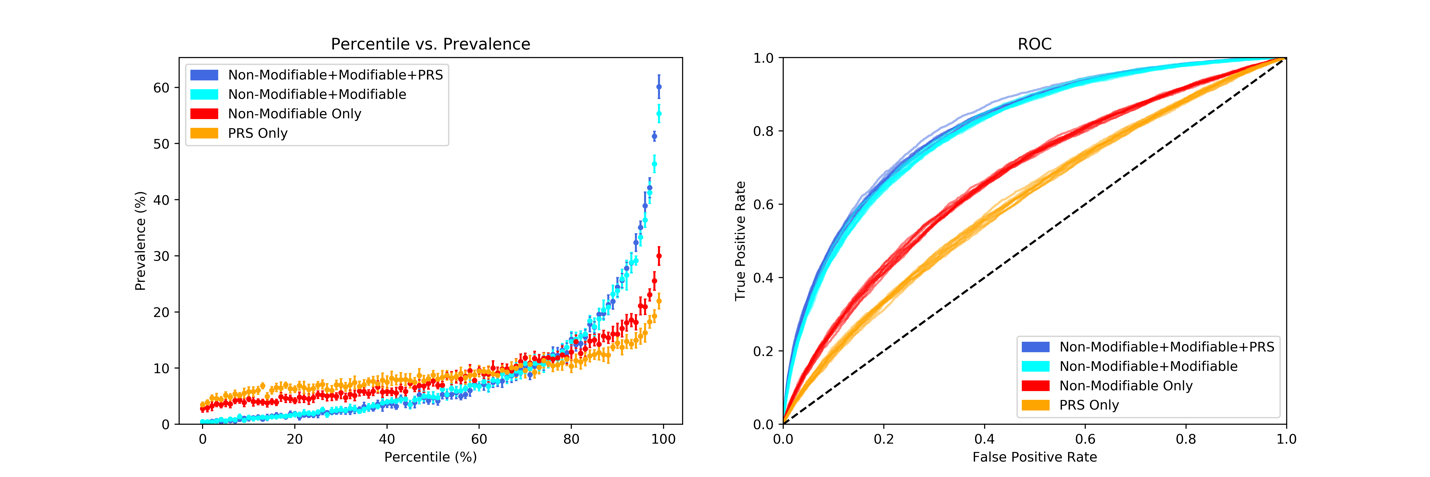
**

**Figure S3. Prevalence vs. Risk Percentile plots and ROC curves for IHD**. The yellow and red curve show how inclusion of sex and genotypic PCs improves on the polygenic risk score model, while the blue curves show the vast improvement upon inclusion of the non-fixed variables. Elevation of risk prediction with PRS (dark blue) is seen only in the top two percentiles relative to the full model not including the PRS (light blue). There is not significant improvement in the area under the curve either.


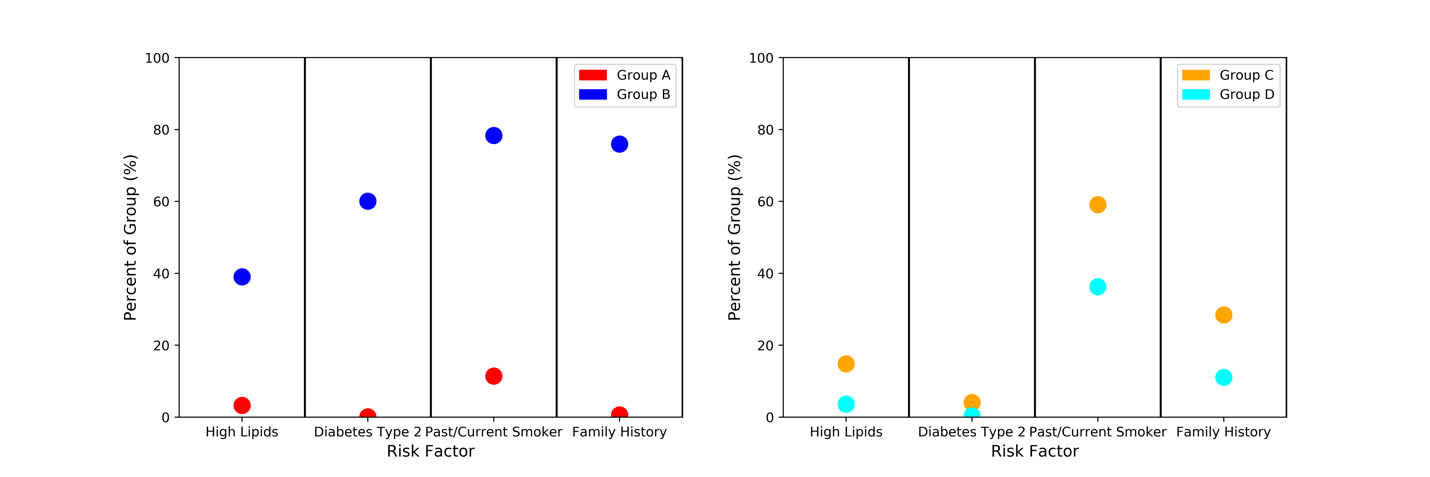


**Figure S4. Distribution of categorical risk factors by non-fixed risk group.**  Comparison of the percent of individuals in each of the non-fixed risk groups from **Figure 5A** who are categorized as having high serum lipid levels, type 2 diabetes, are present smokers, or have a family history of heart disease. Each of these categorical risk factors strongly differentiates the Groups A and B of individuals whose lifetime risk differs from the baseline genetic risk. The high lipids feature in this analysis was taken from UKB Hospital Episodes Statistics (HES) data field 41270, which contains ICD-10 codes for hospital inpatient diagnoses. Any individual with ICD-10 codes E78.0 (pure hypercholesterolemia), E78.1 (pure hyperglyceridemia), or E78.5 (hyperlipidemia) for any of those data fields was coded as “1” and otherwise as “0”.


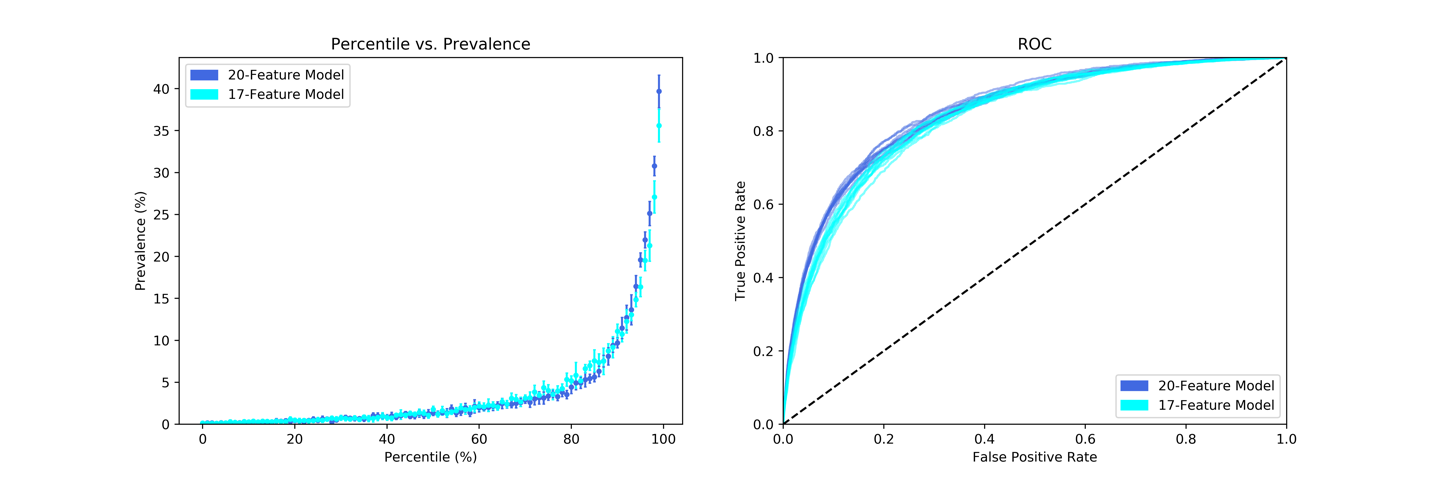


**Figure S5. Prevalence vs. Risk Percentile plots and ROC curves for models including medication use.** This figure compares the full 17-feature model that includes PRS, established-at-birth, and non-fixed features, to a 20-feature model that includes in addition three binary features: taking cholesterol-lowering medications; taking blood pressure-lowering medications; taking neither cholesterol-lowering nor blood pressure-lowering medications.


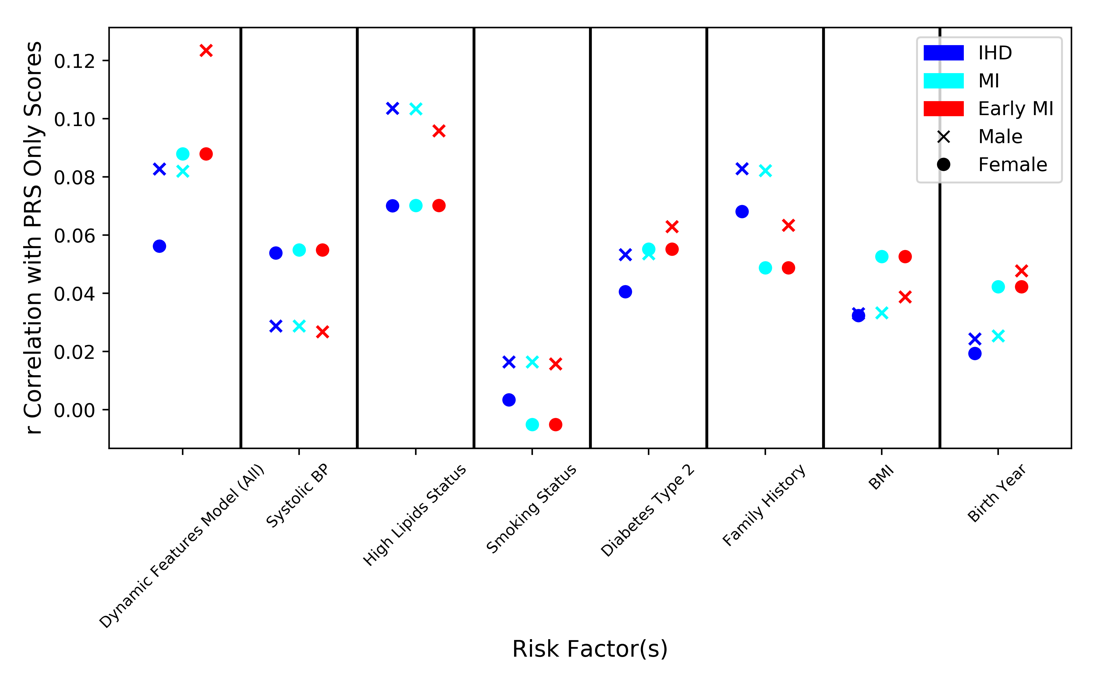


**Figure S6. Correlations between PRS only scores and non-fixed features.** Each cross (males) or dot (females) shows the Pearson correlation between the PRS-only model, 7 non-fixed risk factors, and a combined “dynamic features” model (containing all 7 features listed in the figure; note that lipids includes triglycerides and cholesterol), for the three cardiovascular outcomes. Point biserial r correlations were assessed between the PRS-only model and 6 binary non-fixed risk factors (excluding smoking, which was not binary). The high lipids feature in this analysis was taken from UKB Hospital Episodes Statistics (HES) data field 41270, which contains ICD-10 codes for hospital inpatient diagnoses. Any individual with ICD-10 codes E78.0 (pure hypercholesterolemia), E78.1 (pure hyperglyceridemia), or E78.5 (hyperlipidemia) for any of those data fields was coded as “1” and otherwise as “0”.

1. **B.**


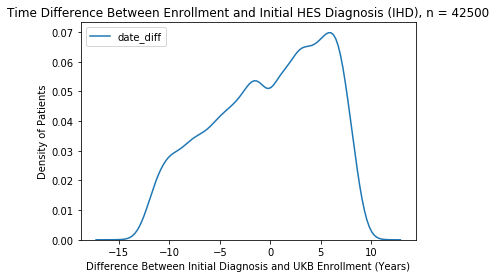

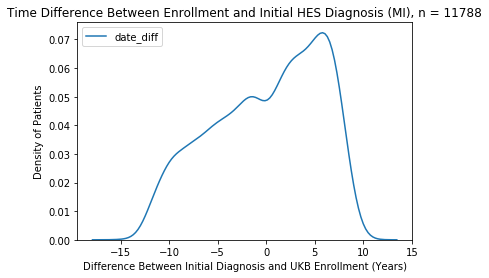


**Figure S7. Time between enrolment and diagnosis in the UK Biobank**. (A) Individuals diagnosed with IHD. (B) Individuals diagnosed with MI. The frequency distributions capture time-to-event after enrolment for incident disease (positive values), and best estimate timing of the diagnosis prior to enrolment in the study (negative values). Number of diagnoses is indicated above each plot.
