# Supplementary material for: Highly elevated polygenic risk scores are better predictors of myocardial infarction risk early in life than later
Source: Genome Med. 2021 Jan 28;13:13. doi: 10.1186/s13073-021-00828-8 (PMC7845089; doi:10.1186/s13073-021-00828-8)
Supplement: Supplementary file 2 — Additional file 2: Supplementary Tables. Table S1. Description of the features used to train logistic regression algorithm. Table S2. Classification of ischemic heart disease (IHD) cases. Table S3. Classification of myocardial infarction (MI) cases. Table S4. Sample sizes for cross-validation algorithm for European-only analyses. Table S5. Total number of individuals in each category of medication intake. Table S6. List of UKB field 20,003 medication codes used for “Blood Pressure Medication - Any” classification for propensity matching analysis. Table S7. List of UKB field 20,003 medication codes used for “Cholesterol Medication - Statins” classification for propensity matching analysis. Table S8. AUCs for univariate logistic regression with individual compared to joint PRS. Table S9. AUCs for multivariate logistic regression with different feature combinations. Table S10. Feature weights from the Established-at-Birth plus PRS (9-Feature) models. Table S11. Feature descriptions for three additional features included in the 20-Feature models for IHD, MI, Early MI. Table S12. Percentile vs. prevalence statistics for multivariate logistic regression for 20-feature model. Table S13. Feature weights from the 20-Feature models for IHD, MI, Early MI. Table S14. AUCs for multivariate logistic regression with different feature combinations, but using categorical hyperlipidemia in place of cholesterol, TG, and LDL measurements. [file 13073_2021_828_MOESM2_ESM.docx]

**ADDITIONAL FILE 2: SUPPLEMENTARY TABLES**

**Table S1**. **Description of the features used to train logistic regression algorithm**

| **Feature Name** | **Feature Description** | **Feature Type** | **Feature Category** |
| --- | --- | --- | --- |
| GRS46K | Polygenic Risk Score | Numerical | PRS |
| FDR202 | Polygenic Risk Score | Numerical | PRS |
| 1.7M | Polygenic Risk Score | Numerical | PRS |
| 6M | Polygenic Risk Score | Numerical | PRS |
| PCs 1-4 | The first four principal components, taken from available UKB genotyping quality control data, used as proxies for ancestry | Numerical | Established-at-birth |
| Birth Year | This was obtained from UKB data field 34 (“Year of Birth”) | Numerical | Established-at-birth |
| Sex | UKB data field 31 - sex classification, acquired from the central registry. Males coded as “1” and females coded as “0”. | Categorical (Binary) | Established-at-birth |
| Systolic Blood Pressure | This information was taken from UKB data fields 4080 and 93. Data from field 4080 consists of systolic blood pressure measurement taken via automated reading using an Omron blood pressure monitor, measured in mmHg, at the first of three assessment visits. Data from field 93 consists of blood pressure measurement taken via manual sphygmometer, at the first of three assessment visits. Each individual had systolic blood pressure data available for either data field 4080, or data field 93. Change over time is not taken into consideration for this analysis. Values for individuals for which systolic blood pressure data was not available were imputed by including the weighted average of all known data points for this feature as the value for each individual for which the data was not available. | Numerical | Non-fixed |
| Cholesterol | This information was taken from UKB data field 30690, which contains blood cholesterol levels for each patient. For this study, the measurements were taken only from the baseline assessment visit. Missing values were imputed as the mean of all available data. | Numerical | Non-fixed |
| Triglycerides | This information was taken from UKB data field 30870, which contains blood triglyceride levels for each patient. The measurements were taken only from the baseline assessment visit and missing values were imputed as the mean of all available data. | Numerical | Non-fixed |
| Type II Diabetes Status | This information was taken from UKB Hospital Episodes Statistics (HES) data field 41270, which contains ICD-10 codes for hospital inpatient diagnoses. Any individual with ICD-10 code E11 (type II diabetes) was coded as “1” and otherwise as “0”. | Categorical (Binary) | Non-fixed |
| Smoking Status | This information was obtained from UKB data field 20116. Individuals who were self-reported as being “Current” smokers in at least one of three assessment visits, and who did not also select “Previous” , “Never”, or “Prefer not to answer” on any other assessment visit, were coded as “2” for this feature. Individuals who were self-reported as being “Previous” smokers on at least one of the assessment visits and who did not also select “Current”, “Never”, or “Prefer not to answer” on any other assessment visit, were coded as “1”. Individuals who were self-reported as “Never” smokers on at least one of the assessment visits and who did not also select “Current”, “Previous”, or “Prefer not to answer” on any other visit, were coded as “0”. The feature value for the remaining individuals was imputed as the weighted average value of the rest of the dataset for this feature. | Categorical (Trinary) | Non-fixed |
| Family History of Heart Disease | This information was obtained from both ICD-10 codes and self-reported data. Any individual with ICD-10 code Z82.4 (Family history of ischemic heart disease and other diseases of the circulatory system) in UKB Hospital Episodes Statistics (HES) data field 41270 was coded as “1”. In addition, UKB data fields 20107, 20110, and 20111, which represent self-reported illnesses of father, mother, and siblings, respectively, were also used. Each of these data fields is based on a touchscreen questionnaire administered at two different assessment visits. If an individual indicated that a family member had “Heart Disease” in at least one assessment visit, the individual was coded as “1” for this feature; otherwise, coded as “0”. | Categorical (Binary) | Non-fixed |
| Body Mass Index (BMI) | This information was taken from UKB data field 21001, which represents the BMI for each patient, measured in kg/m^2^. Only values from the first of three assessment visits were included. Change over time is not taken into consideration for this analysis. Values for individuals for which BMI data was not available were imputed by including the weighted average of all known data points for this feature as the value for each individual for which the data was not available. | Numerical | Non-fixed |

Note: The “Taking Cholesterol-Lowering Medications” and “Taking Blood Pressure-Lowering Medications” features are included in this list because an additional model was later run including these. However, they are not part of the main results.

**Table S2**: **Classification of ischemic heart disease (IHD) cases**

| **Data Field 6150: Vascular/Heart Problems Diagnosed by Doctor**  “Heart Attack” self-reported in at least one of three assessment visits |
| --- |
| **Data Field 20002: Non-Cancer Illness Code, Self-Reported**  “Heart attack/myocardial infarction” self-reported in at least one of three assessment visits |
| **Data Field 20004: Operations Code, Self-Reported**  **“**Coronary angioplasty (PTCA) +/- stent”, “Coronary artery bypass graft (CABG)”, or “Triple heart bypass” self-reported in at least one of three assessment visits |
| **ICD-10 codes I20: Angina Pectoris**  I20.0 Unstable angina I20.8 Other forms of angina pectoris  I20.1 Angina pectoris with documented spasm I20.9 Angina pectoris, unspecified |
| **ICD-10 codes I21: Acute Myocardial Infarction, including:**  I21.0 Acute transmural myocardial infarction of anterior wall  I21.1 Acute transmural myocardial infarction of inferior wall  I21.2 Acute transmural myocardial infarction of other sites  I21.3 Acute transmural myocardial infarction of unspecified site  I21.4 Acute subendocardial myocardial infarction  I21.9 Acute myocardial infarction, unspecified |
| **ICD-10 codes I22: Subsequent Myocardial Infarction, including:**  I22.0 Subsequent myocardial infarction of anterior wall  I22.1 Subsequent myocardial infarction of inferior wall  I22.8 Subsequent myocardial infarction of other sites  I22.9 Subsequent myocardial infarction of unspecified site |
| **ICD-10 codes I23: Certain Complications Following Acute Myocardial Infarction, including:**  I23.0 Haemopericardium as current complication following acute myocardial infarction  I23.1 Atrial septal defect as current complication following acute myocardial infarction  I23.2 Ventricular septal defect as current complication following acute myocardial infarction  I23.3 Rupture of cardiac wall without haemopericardium as current complication following acute MI  I23.5 Rupture of papillary muscle as current complication following acute myocardial infarction  I23.6 Thrombosis of atrium, auricular appendage and ventricle as current complications following acute MI  I23.8 Other current complications following acute myocardial infarction |
| **ICD-10 codes I24: Other Acute Ischemic Heart Disease**  I24.0 Coronary thrombosis not resulting in myocardial infarction  I24.8 Other forms of acute ischaemic heart disease  I24.9 Acute ischaemic heart disease, unspecified |
| **ICD-10 codes I25: Chronic Ischemic Heart Disease**  I25.0 Atherosclerotic cardiovascular disease, so described  I25.1 Atherosclerotic heart disease  I25.2 Old myocardial infarction  I25.5 Ischaemic cardiomyopathy  I25.6 Silent myocardial ischaemia  I25.9 Chronic ischaemic heart disease, unspecified |
| **ICD-10 codes Z95: Presence of Cardiac and Vascular Implants and Grafts**  Z95.1 Presence of aortocoronary bypass graft  Z95.5 Presence of coronary angioplasty implant and graft |

**Table S3**: **Classification of myocardial infarction (MI) cases**

| **Data Field 6150: Vascular/Heart Problems Diagnosed by Doctor**  “Heart Attack” self-reported in at least one of three assessment visits |
| --- |
| **Data Field 20002: Non-Cancer Illness Code, Self-Reported**  “Heart attack/myocardial infarction” self-reported in at least one of three assessment visits |
| **ICD-10 codes I21: Acute Myocardial Infarction, including:**  I21.0 Acute transmural myocardial infarction of anterior wall  I21.1 Acute transmural myocardial infarction of inferior wall  I21.2 Acute transmural myocardial infarction of other sites  I21.3 Acute transmural myocardial infarction of unspecified site  I21.4 Acute subendocardial myocardial infarction  I21.9 Acute myocardial infarction, unspecified |
| **ICD-10 codes I22: Subsequent Myocardial Infarction, including:**  I22.0 Subsequent myocardial infarction of anterior wall  I22.1 Subsequent myocardial infarction of inferior wall  I22.8 Subsequent myocardial infarction of other sites  I22.9 Subsequent myocardial infarction of unspecified site |
| **ICD-10 codes I23: Certain Complications Following Acute Myocardial Infarction, including:**  I23.0 Haemopericardium as current complication following acute myocardial infarction  I23.1 Atrial septal defect as current complication following acute myocardial infarction  I23.2 Ventricular septal defect as current complication following acute myocardial infarction  I23.3 Rupture of cardiac wall without haemopericardium as current complication following acute MI  I23.5 Rupture of papillary muscle as current complication following acute myocardial infarction  I23.6 Thrombosis of atrium, auricular appendage and ventricle as current complications following acute MI  I23.8 Other current complications following acute myocardial infarction |

**Table S4. Sample sizes for cross-validation algorithm for European-only analyses**

|  | **IHD Model** | **MI Model** | **Early MI Model** |
| --- | --- | --- | --- |
| **Full Dataset** | 441,101 total samples  (39,516 cases,  401,585 controls) | 441,101 total samples  (15,930 cases,  425,171 controls) | 441,101 total samples  (669 cases,  440,432 controls) |
| **Unbalanced Training Set (90% of total dataset)** | ~396,991 total samples  (~35,564 cases, ~361,427 controls) | ~396,991 total samples (~14,337 cases, ~382,654 controls) | ~396,991 total samples  (~602 cases,  ~396,389 controls) |
| **Balanced Training Set (Used for Training LR)** | ~71,128 total samples  (~35,564 cases, ~35,564 controls) | ~37,904 total samples  (~14,337 cases, ~14,337 controls) | ~1,206 total samples  (~602 cases,  ~602 controls) |
| **Test Set (10% of total dataset)** | ~44,110 total samples  (~3952 cases,  ~40,158 controls) | ~44,110 total samples  (~1,593 cases,  ~42,517 controls) | ~44,110 total samples  (~67 cases,  ~44,043 controls) |

**Table S5**. **Total number of individuals in each category of medication intake**

|  | **Total Number of Individuals** |
| --- | --- |
| **Taking Blood Pressure Medications and Not Taking Statins** | 34,015 |
| **Taking Statins and Not Taking Blood Pressure Medications** | 8,672 |
| **Taking Neither Statins Nor Blood Pressure Medications** | 398,313 |

This is not based on using data field 6177, but is instead based on analysis of data field 20003 medication codes

**Table S6**. **List of UKB field 20003 medication codes used for**

**“Blood Pressure Medication - Any” classification for propensity matching analysis**

| **Ace Inhibitors** | **Angiotensin II Antagonists** | **Beta Blockers** | **Calcium Channel Blockers** | **Centrally Acting Alpha Adrenergics** | **Peripherally Acting Alpha Adrenergic Blockers** | **Vasodilators** | **Combination Drugs** |
| --- | --- | --- | --- | --- | --- | --- | --- |
| lisinopril | losartan | nadolol | atenolol+nifedipine 50mg/20mg m/r capsule | catapres 100mcg tablet | hytrin 2mg tablet | minoxidil | tarka 2mg/180mg m/r capsule |
| zestril 2.5mg tablet | cozaar half strength 25mg tablet | corgard 40mg tablet | nifedipine | clonidine hydrochloride 25micrograms tablet | cardura 1mg tablet | hydralazine |  |
| quinapril | valsartan | trandate 50mg tablet | cardene 20mg capsule | apraclonidine | doxazosin |  |  |
| captopril | diovan 40mg capsule | pindolol | isradipine | clonidine | phenoxybenzamine |  |  |
| capoten 12.5mg tablet | losartan potassium+hydrochlorothiazide 50mg/12.5mg tablet | metoprolol tartrate+chlorthalidone 100mg/12.5mg tablet | nimodipine |  | prazosin |  |  |
| captopril+hydrochlorothiazide 25mg/12.5mg tablet | cozaar-comp 50mg/12.5mg tablet | nadolol+bendrofluazide 40mg/5mg tablet | amlodipine |  | terazosin |  |  |
| enalapril maleate+hydrochlorothiazide 20mg/12.5mg tablet | irbesartan | nadolol+bendrofluazide 80mg/5mg tablet | diltiazem |  |  |  |  |
| ramipril | candesartan cilexetil | penbutolol sulphate+frusemide 40mg/20mg tablet | nicardipine |  |  |  |  |
| trandolapril | telmisartan | pindolol+clopamide 10mg/5mg tablet | verapamil |  |  |  |  |
| lisinopril+hydrochlorothiazide 10mg/12.5mg tablet | eprosartan | timolol maleate+co-amilozide 10mg/2.5mg/25mg tablet | felodipine |  |  |  |  |
| enalapril | teveten 300mg tablet | timolol maleate+bendrofluazide 10mg/2.5mg tablet | calanif 5mg capsule |  |  |  |  |
| fosinopril | micardis 20mg tablet | timolol maleate+bendrofluazide 20mg/5mg tablet | diltiazem hcl+hydrochlorothiazide 150mg/12.5mg m/r capsule |  |  |  |  |
| perindopril | irbesartan+hydrochlorothiazide 150mg/12.5mg tablet | metoprolol tartrate+hydrochlorothiazide 100mg/12.5mg tablet | plendil 2.5mg m/r tablet |  |  |  |  |
| moexipril | cozaar 25mg tablet | propranolol hydrochloride+bendrofluazide 80mg/2.5mg capsule | nisoldipine |  |  |  |  |
| trandolapril+verapamil hydrochloride | telmisartan+hydrochlorothiazide 40mg/12.5mg tablet | acebutolol+hydrochlorothiazide 200mg/12.5mg tablet | trandolapril+verapamil hydrochloride |  |  |  |  |
| felodipine+ramipril | micardisplus 40mg/12.5mg tablet | atenolol+nifedipine 50mg/20mg m/r capsule | felodipine+ramipril |  |  |  |  |
| perindopril+indapamide | olmesartan | bisoprolol fumarate+hydrochlorothiazide 10mg/6.25mg tablet |  |  |  |  |  |
|  | valsartan+hydrochlorothiazide 80mg/12.5mg tablet | acebutolol |  |  |  |  |  |
|  | co-diovan 80mg/12.5mg tablet | sectral 100mg capsule |  |  |  |  |  |
|  |  | atenolol |  |  |  |  |  |
|  |  | tenormin 25 tablet |  |  |  |  |  |
|  |  | half-inderal la 80mg m/r capsule |  |  |  |  |  |
|  |  | inderal 10mg tablet |  |  |  |  |  |
|  |  | timolol 0.25% eye drops |  |  |  |  |  |
|  |  | betaxolol |  |  |  |  |  |
|  |  | bisoprolol |  |  |  |  |  |
|  |  | metoprolol |  |  |  |  |  |
|  |  | labetalol |  |  |  |  |  |
|  |  | penbutolol |  |  |  |  |  |
|  |  | propranolol |  |  |  |  |  |
|  |  | timolol |  |  |  |  |  |
|  |  | carvedilol |  |  |  |  |  |
|  |  | atenolol+chlorthalidone |  |  |  |  |  |
|  |  | atenolol+bendrofluazide |  |  |  |  |  |
|  |  | atenolol+co-amilozide |  |  |  |  |  |
|  |  | nebivolol |  |  |  |  |  |
|  |  | dorzolamide+timolol |  |  |  |  |  |
|  |  | atenolol+chlortalidone |  |  |  |  |  |
|  |  | latanoprost+timolol |  |  |  |  |  |
|  |  | nadolol+bendroflumethiazide 40mg/5mg tablet |  |  |  |  |  |
|  |  | timolol maleate+bendroflumethiazide 10mg/2.5mg tablet |  |  |  |  |  |
|  |  | atenolol+bendroflumethiazide |  |  |  |  |  |

**Table S7.**  **List of UKB field 20003 medication codes used for**

**“Cholesterol Medication - Statins” classification for propensity matching analysis**

| Code | Medication |
| --- | --- |
| 1140861958 | simvastatin |
| 1140864592 | lescol 20mg capsule |
| 1140881748 | zocor 10mg tablet |
| 1140888594 | fluvastatin |
| 1140888648 | pravastatin |
| 1141146138 | lipitor 10mg tablet |
| 1141146234 | atorvastatin |
| 1141192410 | rosuvastatin |
| 1141192414 | crestor 10mg tablet |
| 1141200040 | zocor heart-pro 10mg tablet |

**Table S8. AUCs for univariate logistic regression with individual compared to joint PRS**

|  | **FDR202** | **GRS46K** | **1.7M** | **6M** | **All PRS** |
| --- | --- | --- | --- | --- | --- |
| **Ischemic Heart Disease (IHD)** | 0.57 | 0.57 | 0.58 | 0.60 | 0.61 |
| **Myocardial Infarction (MI)** | 0.60 | 0.59 | 0.60 | 0.63 | 0.64 |
| **Early MI** | 0.65 | 0.64 | 0.66 | 0.68 | 0.70 |

95% confidence intervals of AUC from 10-fold cross-validation are ± 0.00 for IHD and MI, or ± 0.02 for Early MI.

**Table S9. AUCs for multivariate logistic regression with different feature combinations**

|  | **PRS Only**  **(4-Feature Model)** | **Established-at-birth**  **(5-Feature Model)** | **Established-at-birth plus PRS**  **(9-Feature Model)** | **Established-at-birth plus non-fixed**  **(13-Feature Model)** | **Established-at-birth plus non-fixed plus PRS**  **(17-Feature Model)** | **20-Feature Model including Medication** |
| --- | --- | --- | --- | --- | --- | --- |
| **Ischemic Heart Disease (IHD)** | 0.61 | 0.62 | 0.67 | 0.80 | 0.81 | 0.83 |
| **Myocardial Infarction (MI)** | 0.64 | 0.67 | 0.73 | 0.83 | 0.84 | 0.85 |
| **Early MI** | 0.70 | 0.70 | 0.79 | 0.92 | 0.93 | 0.95 |

95% confidence intervals of AUC from 10-fold cross-validation are ± 0.00 for IHD and MI, or ± 0.01 - 0.02 for Early MI. The 20-Feature model includes all the features of the 17-Feature model, as well as three additional binary features: 1) taking cholesterol-lowering medications [1/0], and 2) taking blood pressure-lowering medications [1/0], 3) taking neither cholesterol-lowering nor blood pressure-lowering medications [1/0].

**Table S10. Feature weights from the Established-at-birth plus PRS (9-Feature) models**

| **Feature** | **Weight**  **(IHD Score)** | **Weight**  **(MI Score)** | **Weight**  **(Early MI Score)** |
| --- | --- | --- | --- |
| **FDR202** | 0.09 +/- 0.01 | 0.14 +/- 0.00 | 0.30 +/-0.02 |
| **GRS46K** | 0.20 +/- 0.00 | 0.26 +/- 0.01 | 0.30 +/- 0.02 |
| **1.7M** | 0.16 +/- 0.01 | 0.22 +/- 0.00 | 0.51 +/- 0.02 |
| **6M** | 1.62 +/- 0.00 | 1.79 +/- 0.06 | 0.13 +/- 0.03 |
| **Gender** | 0.96 +/- 0.00 | 1.41 +/- 0.00 | 1.91 +/- 0.04 |
| **PC1** | -0.03 +/- 0.05 | 0.04 +/-0.06 | -0.01 +/- 0.38 |
| **PC2** | 0.17 +/- 0.03 | 0.17 +/- 0.05 | -0.03 +/- 0.02 |
| **PC3** | 0.01 +/- 0.00 | 0.02 +/- 0.00 | 0.03 +/- 0.01 |
| **PC4** | 0.002 +/- 0.00 | 0.01 +/- 0.00 | 0.01 +/- 0.00 |

**Table S11. Feature descriptions for three additional features included in the 20-Feature models for IHD, MI, Early MI**

| **Feature Name** | **Feature Description** | **Feature Type** | **Feature Category** |
| --- | --- | --- | --- |
| Taking Cholesterol-Lowering Medications | This information was taken from UKB data field 6177. Individuals taking cholesterol-lowering medications but not blood pressure-lowering medications were coded as ‘1’, and those taking both, neither, or only blood pressure-lowering medications were coded as ‘0’. The values for the remaining individuals were imputed as the mean of the known values | Categorical (Binary) | Modifiable |
| Taking Blood Pressure-Lowering Medications | This information was taken from UKB data field 6177. Individuals taking blood pressure-lowering medications but not cholesterol-lowering medications were coded as ‘1’, and those taking both, neither, or only cholesterol-lowering medications were coded as ‘0’. The values for the remaining individuals were imputed as the mean of the known values. | Categorical (Binary) | Modifiable |
| Taking Neither Cholesterol-Lowering nor Blood Pressure-Lowering Medications | This information was taken from UKB data field 6177. Individuals taking neither blood pressure-lowering medications nor cholesterol-lowering medications were coded as ‘1’, and those taking just one or neither were coded as ‘0’. The values for the remaining individuals were imputed as the mean of the known values. | Categorical (Binary) | Modifiable |

**Table S12. Percentile vs. prevalence statistics for multivariate logistic regression for 20-feature model**

| **Percentile** | **IHD** | **MI** | **Early MI** |
| --- | --- | --- | --- |
| **Top Percentile** | 64.81% +/- 1.45 | 39.68 +/- 1.94 | 7.23% +/- 0.83 |
| **Middle Percentile** | 4.62% +/- 0.43 | 1.34 +/- 0.30 | 0.00% +/- 0.00 |
| **Lowest Percentile** | 0.39% +/- 0.19 | 0.09 +/- 0.12 | 0.00% +/- 0.00 |

**Table S13. Feature weights from the 20-Feature models for IHD, MI, Early MI**

| **Feature** | **Weight**  **(IHD Score)** | **Weight**  **(MI Score)** | **Weight**  **(Early MI Score)** |
| --- | --- | --- | --- |
| **Taking Cholesterol-Lowering Medications** | -0.55 +/- 0.01 | -0.69 +/- 0.01 | -0.72+/-0.05 |
| **Taking Blood Pressure-Lowering Medications** | -1.04 +/- 0.01 | -1.20 +/- 0.01 | -2.12 +/- 0.08 |
| **Taking Neither Cholesterol-Lowering nor Blood Pressure-Lowering Medications** | -1.58 +/- 0.01 | -1.72 +/- 0.01 | -3.52 +/- 0.07 |

**Table S14. AUCs for multivariate logistic regression with different feature combinations, but using categorical hyperlipidemia in place of cholesterol, TG, and LDL measurements.**

|  | **Established-at-birth plus Non-fixed**  **(12-Feature Model)** | **Established-at-birth plus Non-fixed**  **plus PRS**  **(16-Feature Model)** |
| --- | --- | --- |
| **Ischemic Heart Disease (IHD)** | 0.85 | 0.85 |
| **Myocardial Infarction (MI)** | 0.86 | 0.87 |
| **Early MI** | 0.94 | 0.94 |

95% confidence intervals of AUC from 10-fold cross-validation are ± 0.00 for IHD and MI, or ± 0.01 - 0.02 for Early MI. Compared with results in Additional Table S9 for continuous lipid measurement, more variance is explained with categorical hyperlipidemia.
